# Development of eSSR-Markers in *Setaria italica* and Their Applicability in Studying Genetic Diversity, Cross-Transferability and Comparative Mapping in Millet and Non-Millet Species

**DOI:** 10.1371/journal.pone.0067742

**Published:** 2013-06-21

**Authors:** Kajal Kumari, Mehanathan Muthamilarasan, Gopal Misra, Sarika Gupta, Alagesan Subramanian, Swarup Kumar Parida, Debasis Chattopadhyay, Manoj Prasad

**Affiliations:** 1 National Institute of Plant Genome Research, New Delhi, India; 2 Tamil Nadu Agricultural University, Coimbatore, India; Georgia Institute of Technology, United States of America

## Abstract

Foxtail millet ( *Setaria* *italica* L.) is a tractable experimental model crop for studying functional genomics of millets and bioenergy grasses. But the limited availability of genomic resources, particularly expressed sequence-based genic markers is significantly impeding its genetic improvement. Considering this, we attempted to develop EST-derived-SSR (eSSR) markers and utilize them in germplasm characterization, cross-genera transferability and *in silico* comparative mapping. From 66,027 foxtail millet EST sequences 24,828 non-redundant ESTs were deduced, representing ~16 Mb, which revealed 534 (~2%) eSSRs in 495 SSR containing ESTs at a frequency of 1/30 kb. A total of 447 pp were successfully designed, of which 327 were mapped physically onto nine chromosomes. About 106 selected primer pairs representing the foxtail millet genome showed high-level of cross-genera amplification at an average of ~88% in eight millets and four non-millet species. Broad range of genetic diversity (0.02–0.65) obtained in constructed phylogenetic tree using 40 eSSR markers demonstrated its utility in germplasm characterizations and phylogenetics. Comparative mapping of physically mapped eSSR markers showed considerable proportion of sequence-based orthology and syntenic relationship between foxtail millet chromosomes and sorghum (~68%), maize (~61%) and rice (~42%) chromosomes. Synteny analysis of eSSRs of foxtail millet, rice, maize and sorghum suggested the nested chromosome fusion frequently observed in grass genomes. Thus, for the first time we had generated large-scale eSSR markers in foxtail millet and demonstrated their utility in germplasm characterization, transferability, phylogenetics and comparative mapping studies in millets and bioenergy grass species.

## Introduction

Foxtail millet [ *Setaria* *italica* (L.) P. Beauv.] possesses a small genome (~515Mb; 2n=2x=18) with a relatively lower repetitive DNA and its inbreeding nature coupled to short life-cycle has accentuated this crop as an experimental model system to decipher architectural traits, evolutionary genomics and physiological aspects of C_4_ panicoid grass crops [1,2]. Remarkably, the close proximity of foxtail millet to various biofuel crops namely, switchgrass ( *Panicum* *virgatum* ), napiergrass ( *Pennisetum* *purpureum* ) and pearl millet ( *Pennisetum* *glaucum* ) which are a difficult target for whole genome sequencing, had enabled the crop to serve as an essential substitute genome to exploit these crops [2,3]. Recently, the US Department of Energy-Joint Genome Institute (DOE-JGI) and BGI (formerly the Beijing Genomics Institute), China sequenced the genome of foxtail millet [4,5].

Acquaintance of information on genetic basis of yield, disease resistance and abiotic stress tolerance are vital criteria for formulating breeding strategies for genetic improvement of foxtail millet. Nevertheless, in comparison to other economically important crops, relatively less effort has been invested in deciphering the genetics of important agronomic traits of foxtail millet. Although there are efforts in progress for foxtail millet improvement through conventional breeding, molecular breeding has a greater potential to accelerate the utilization of genetic diversity available in this crop, especially among the land races and related germplasm lines [6]. The identification of molecular markers that are tightly linked to genes/QTLs (quantitative trait loci) controlling the important agronomic and abiotic stress-responsive traits is a prerequisite for undertaking molecular breeding in plants [7-10].

Simple sequence repeat (SSR) markers are randomly distributed within the genome at high abundance with high polymorphism information content and co-dominant inheritance. But genomic SSR markers developed from SSR-enriched genomic libraries or random genomic sequences are derived principally from inter-genic DNA regions, and consequently encompass putative linkage to the transcribed regions of the genome. Conversely, genic-SSR (EST-SSRs or eSSRs) markers exclusively target the transcribed region of the genome and have increased potential for linkage to loci that contribute to agronomic phenotypes. As a result, when polymorphic eSSR markers are identified in high-value breeding lines they can possess significant efficacy for marker assisted selection (MAS) [11,12]. In addition, the eSSR markers reveal improved cross-genome comparisons through cross-transferability and comparative mapping as they target protein-coding regions that are conserved between related species [13].

The present investigation was therefore conducted to (i) develop and characterize EST-derived SSR markers for *S* *. italica* , (ii) develop physical map for *in-silico* genic microsatellite marker-based comparative mapping between foxtail millet and other grass species, (iii) evaluate their potential for cross-genera transferability and (iv) genetic diversity.

## Materials and Methods

### Plant material and DNA isolation

The details of plant materials used in the study are listed in Table S1. The seeds of all the investigated species were surface sterilized in 3% sodium hypochlorite for 20 minutes, rinsed with sterile distilled water and were germinated in greenhouse. The genomic DNA was isolated from the fresh young leaves by CTAB method as described elsewhere [14]. The DNA was purified and then quantified on agarose gel by comparison with 50ng/µl of standard lambda (λ) DNA marker (NEB).

### Database search for eSSRs and primer design

The publicly available EST sequences of *S* *. italica* were searched and retrieved from NCBI dbEST (ftp://ftp.ncbi.nih.gov/blast/db/). Approximately 66,027 ESTs were used for the unigene definition using CD-HIT (Cluster Database at High Identity with Tolerance) software tool (http://weizhong-lab.ucsd.edu/cdhit_suite/cgi-bin/index.cgi) for redundancy minimization and assembling of sequences. Consequently, the assembled sequences were searched for identification and localization of microsatellites (SSRs) by employing MISA (MIcroSAtellite, http://pgrc.ipk-gatersleben.de/misa/) software tool. Based on repeat motifs microsatellites were classified into three categories: perfect (N_1_N_2_)_x_ or (N_1_N_2_N_3_)_x_; interrupted (N_1_N_2_)_x_N_y_ (N_1_N_2_)_z_ and compound (N_1_N_2_)_x_N_y_(N_3_N_4_)_z_; (N_1_N_2_N_3_)_x_(N_1_N_2_)_y_ types [15]. Subsequently, forward and reverse primers from the flanking sequences of eSSRs were designed in batches using the integrated MISA and PRIMER3 Perl5 interface modules. The conditions with 100-300 bp product size in length, optimal melting temperature 55-60^0^C, primer size 20 bases and GC contents from 50 to 70% were used for designing the primers.

### Physical mapping of eSSR markers

The putative functions of the eSSR markers were assigned by comparison with the non-redundant database at NCBI using the BLASTX program (http://blast.ncbi.nlm.nih.gov/Blast.cgi) with default search parameters. The eSSR markers were BLAST searched against the whole genome sequences of foxtail millet available at Phytozome (http://www.phytozome.net) and plotted individually on each of the nine foxtail millet chromosomes according to their ascending order of physical position (bp), from the short arm telomere to the long arm telomere and finally visualized in MapChart software [16].

### eSSR markers amplification and sequence analysis

The eSSRs were amplified in a 25µl total volume containing 1 unit of Taq DNA polymerase (Sigma), 50 ng of genomic DNA, 10µmol/L of each primer, 0.5 mmol/L of each dNTPs, and 2.5µl of 10X PCR reaction buffer (500 mM KCl, 200 mM Tris-HCl [pH 8.4], and 3 mM MgCl_2_] in iCycler thermal controller (Bio-Rad). The PCR profile was: an initial denaturation of 3 min at 94°C, followed by 35 cycles of 60 s at 94°C, 60 s at 50-55°C, and 2 min at 72°C, and a final extension of 10 min at 72°C. The amplicons were resolved on 2% agarose gel (Cambrex, USA) in Tris-borate EDTA (TBE) buffer (pH 8.0), stained with ethidium bromide and analyzed using GelDoc-It^TM^ imaging system (UVP). The fragment size for each locus was determined by 100bp standard size markers (NEB). Results were confirmed by three replicate assays.

The amplified products (alleles) from millet and non-millet species were eluted and cloned into pGEM^(R)^-T Easy vector (Promega) following the manufacturer’s instructions. The recombinant plasmids were purified using *AccuPrep* Plasmid MiniPrep DNA Extraction Kit (Bioneer) following the manufacturer’s protocol. The plasmids were sequenced in automated sequencer (3730xI DNA Analyzer, Applied Biosystems) using M13 forward and reverse primers. Multiple sequence alignment was performed with the obtained sequences along with reference *S* *. italica* sequence using ClustalW2 program (http://www.ebi.ac.uk/Tools/clustalw2/index.html).

### Analysis of genetic relationship

The eSSR markers profiles amplified among foxtail millet accessions were scored manually; each allele was scored as present (1) or absent (0) for each of the eSSR loci. Polymorphic informative content (PIC) were calculated according to Roldán-Ruiz et al. [17] as: PIC_*i*_ = 2*f*_*i*_ *(*1-*f*_*i*_ *)*, where *f*_*i*_ is the frequency of the amplified allele (band present) and (1*-f*_*i*_) is the frequency of the null allele (band absent) of marker *i*. Using pairwise similarity matrix of Jaccard’s coefficient [18], the level of genetic diversity among foxtail millet accessions was calculated and a phylogenetic tree was constructed by unweighted pair-group method of arithmetic average (UPGMA), neighbor-joining (NJoin) module of the NTSYS-pc software v 2.02 [19]. The genetic relationships among millets and non-millet grass species based on cross-transferability of SSR markers was determined based on Nei (1983) diversity co-efficient and phylogenetic tree was constructed using the neighbour-joining (NJ) tree interface of PowerMarker software ver 2.5 [20]. The observed heterozygosity (*H*_*O*_), Nei’s average gene diversity [21], Fixation index (*F*_IS_) and Shannon’s Informative Index (*I*) were computed by POPGENE 1.32 [22]. Correlation analysis among PIC, number of repeat unit and number of alleles were analyzed by GraphPad InStat software v 3.10 (www.graphpad.com).

### *In-silico* comparative genome mapping

The eSSR markers that were physically mapped on the nine chromosomes of foxtail millet were BLASTN searched against genome sequences of sorghum, maize and rice (www.phytozome.net) to develop marker-based syntenic relationships among the chromosomes of foxtail millet and three other grass species. A cut-off bit score of 54.7 and E value of < 1e-05 were considered optimum for BLASTN analysis. The marker-based syntenic relationships among foxtail millet, rice, sorghum and maize were finally visualized with visualization blocks in Circos software v 0.55 (http://circos.ca) [23]. The protein sequences corresponding to the detected anchor points of the collinear regions were aligned using ClustalW. The extracted nucleotide sequences were aligned by PAML4.5 package [24]. Synonymous substitution per synonymous site (Ks) was determined using yn00 tool [25,26].

## Results

### Frequency and distribution of eSSRs in foxtail millet

**Figure 1:**
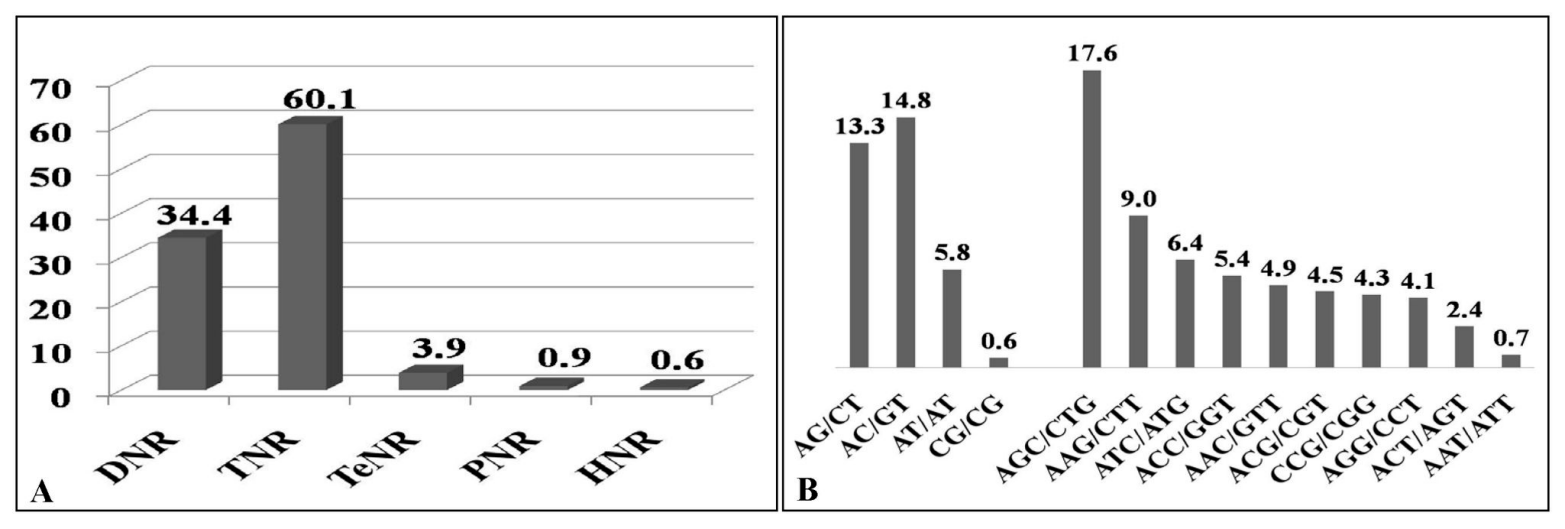
Analysis of simple sequence repeats from foxtail millet genome. (A) Frequency, proportion (%) of microsatellite repeats. (B) Different Di- and tri-nucleotide repeat motif types identified in the foxtail millet genome. DNR - Di-nucleotide repeat; TNR - Tri-nucleotide repeat; TeNR - Tetra-nucleotide repeat; PNR - Penta-nucleotide repeat; HNR - Hexa-nucleotide repeat.

**Figure 2:**
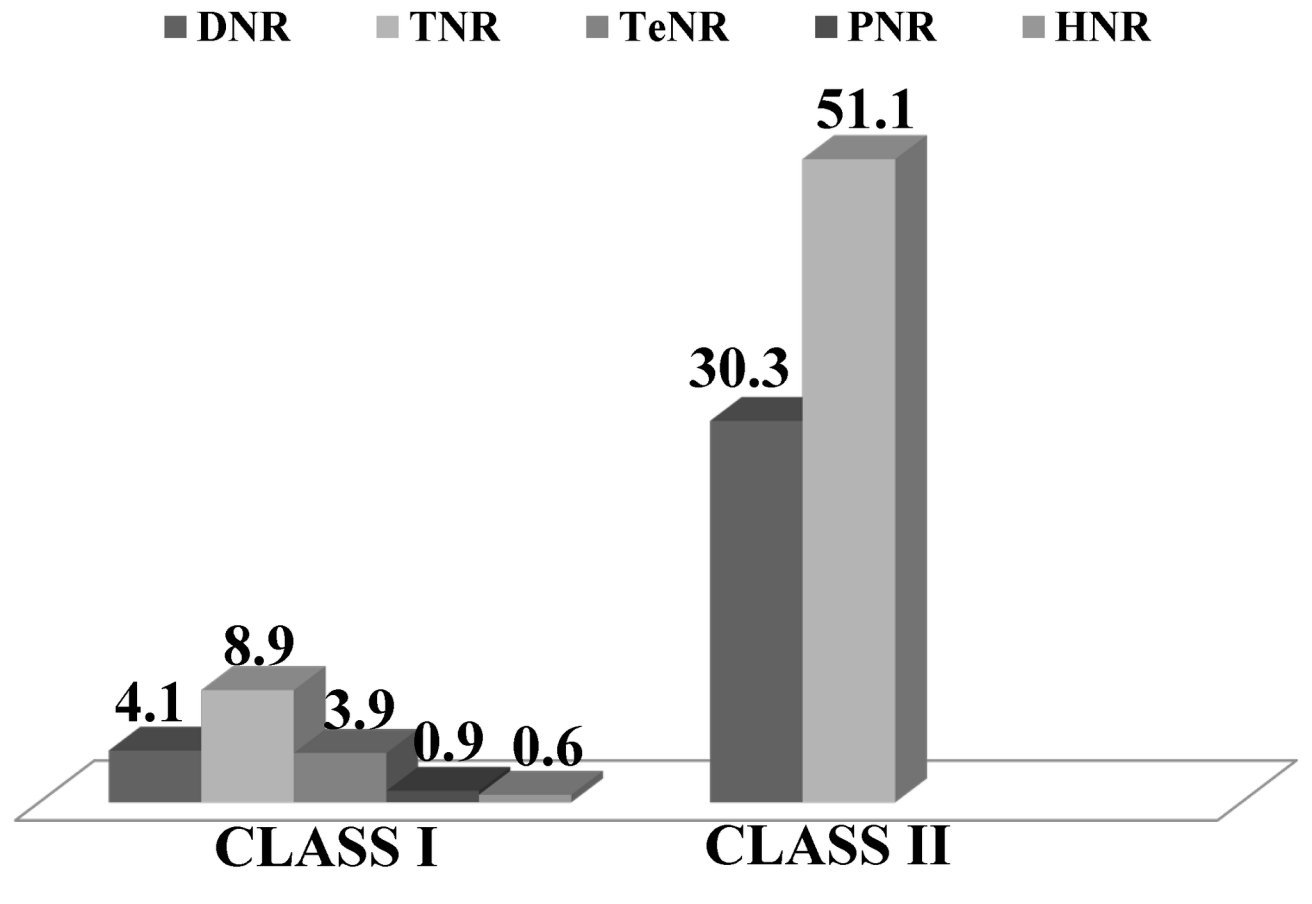
Frequency and relative distribution of long and hypervariable class I and variable class II microsatellite repeats in coding regions of foxtail millet genome. DNR - Di-nucleotide repeat; TNR - Tri-nucleotide repeat; TeNR - Tetra-nucleotide repeat; PNR - Penta-nucleotide repeat; HNR - Hexa-nucleotide repeat.

We examined a set of 66,027 EST sequences of *S* *. italica* and detected 24,828 non-redundant ESTs. The mining of SSRs in 24,828 non-redundant EST sequences representing ~16 Mb revealed the presence of 534 SSRs (~2%) in 495 SSR containing ESTs, of which 35 (7%) contained more than one SSR. This corresponds to an average distance between SSRs of approximately 30 kb or 1 SSR-containing EST in every 50.1 ESTs. Among the total 534 eSSRs, 27 (~5%) appeared to have compound type. The frequency and distribution of 534 non-redundant eSSRs in *S* *. italica* is shown in Table S2. The eSSRs contained diverse types of repeat motifs. The tri-nucleotides were the most frequent, with a frequency of 321 (60.1%), followed by di- 184 (34.4%), tetra- 21 (3.9%), penta- 5 (0.9%) and hexa-nucleotide 3 (0.6%) (Figure 1A). Among the tri-nucleotides, AGC/CTG (17.6%) motifs were most abundant followed by AAG/CTT (9%). Among di-nucleotides, AC/GT motifs (14.8%) were more frequent, followed by AG/CT (13.3%), AT/AT (5.8%) and CG/CG (0.6%) motifs (Figure 1B). Based on length of the repeat-motifs, a total of 99 (18.5%) eSSRs were classified as long and hyper-variable class I (≥ 20bp) types and remaining 435 (81.5%) were classified as variable class II (12-19bp) types (Figure 2). Interestingly, in both class I and class II tri-nucleotides repeat motifs were detected in higher proportion, as in case of class I microsatellites, the proportion of tri-nucleotide (~9%) was higher as compared to di-(4.1%) and-tetra-nucleotide (3.9%). Similarly, in the class II microsatellite types, the proportion of tri-nucleotides (51%) was more than that of di-nucleotides (30.3%) (Figure 2).

### Development and validation of eSSR markers

Out of the 495 SSR containing ESTs, primer pairs (pp) could be successfully designed for 447 (90.3%) SSRs (Table S3). Of the 447 primer pairs, 241 (53.9%) belongs to tri-nucleotide, followed by di-136 (30.4%), compound-32 (7.2%), mono-16 (3.6%), tetra-15 (3.4%) and penta-4 (0.9%) and hexa-nucleotide 3 (0.7%). BLASTX annotation was performed to decipher the putative functions of EST sequences from which the SSR markers were developed. It was found that, about 66 (15.7%) of the 420 SSR containing ESTs were annotated in functional protein-encoding sequences, whereas 354 (84.3%) were putative/ hypothetical/ uncharacterized/ unknown/ no considerable homology (Table S3). All the 447 eSSR markers were submitted in NCBI Probe Database public domain with accession numbers from PUIDs 16719049 to 16719495 (Table S3). Of the 447 pp reported in the present study, only 26 (5.8%) are common as reported in one of our recent study on genomic SSRs [27], and are mentioned in the Table S3.

From the 447 eSSR markers developed, a set of 106 markers were chosen representing the whole genome of *S* *. italica* for validation in cv. Prasad, where all the 106 markers produced clear amplification profiles.

### Physical mapping of developed eSSR markers

**Figure 3:**
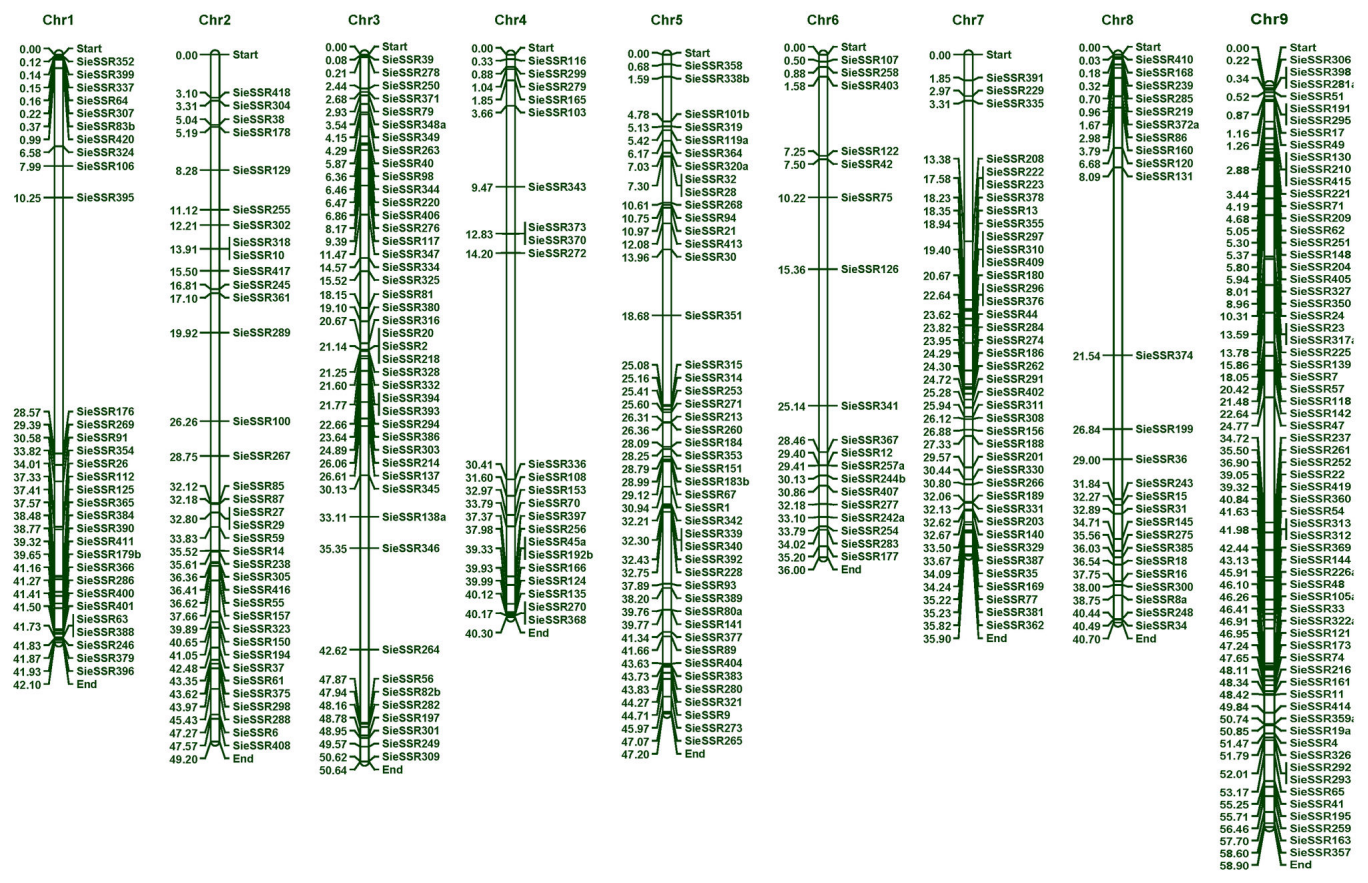
A physical map of foxtail millet based on 327 eSSR markers.

The determination of genomic distribution of 447 eSSR markers on the foxtail millet genome revealed physical localization of 327 markers on the nine chromosomes of foxtail millet with average marker density of 0.8 markers/Mb (Figure 3, Table S3). All the physically mapped 327 eSSR markers were placed in publicly available NCBI Probe Database. The average marker density was maximum (1.12/Mb) in the chromosome 9, followed by chromosome 7 (1.11/Mb) and minimum in the chromosome 6 (0.5/Mb). An extensive analysis of chromosome-wise distribution and frequency of these physically mapped microsatellite markers showed higher frequency of markers mapped on chromosome 9 (66 markers, 20.2%) and minimum on chromosome 6 (18, 5.5%) (Table 1).

**Table 1:** Chromosomal distribution and average density of eSSR markers mapped on the nine chromosomes of foxtail millet.

Chromosome	Markers mapped (%)	Density (markers/Mb)
Chr.1	31 (9.5)	0.74
Chr.2	36 (11)	0.73
Chr.3	44 (13.4)	0.87
Chr.4	22 (6.7)	0.55
Chr.5	45 (13.7)	0.95
Chr.6	18 (5.5)	0.50
Chr.7	40 (12.2)	1.11
Chr.8	25 (7.6)	0.61
Chr.9	66 (20.2)	1.12
**Average**		**0.79**

### Cross-genera transferability and genetic basis of sequence length variation

**Figure 4:**
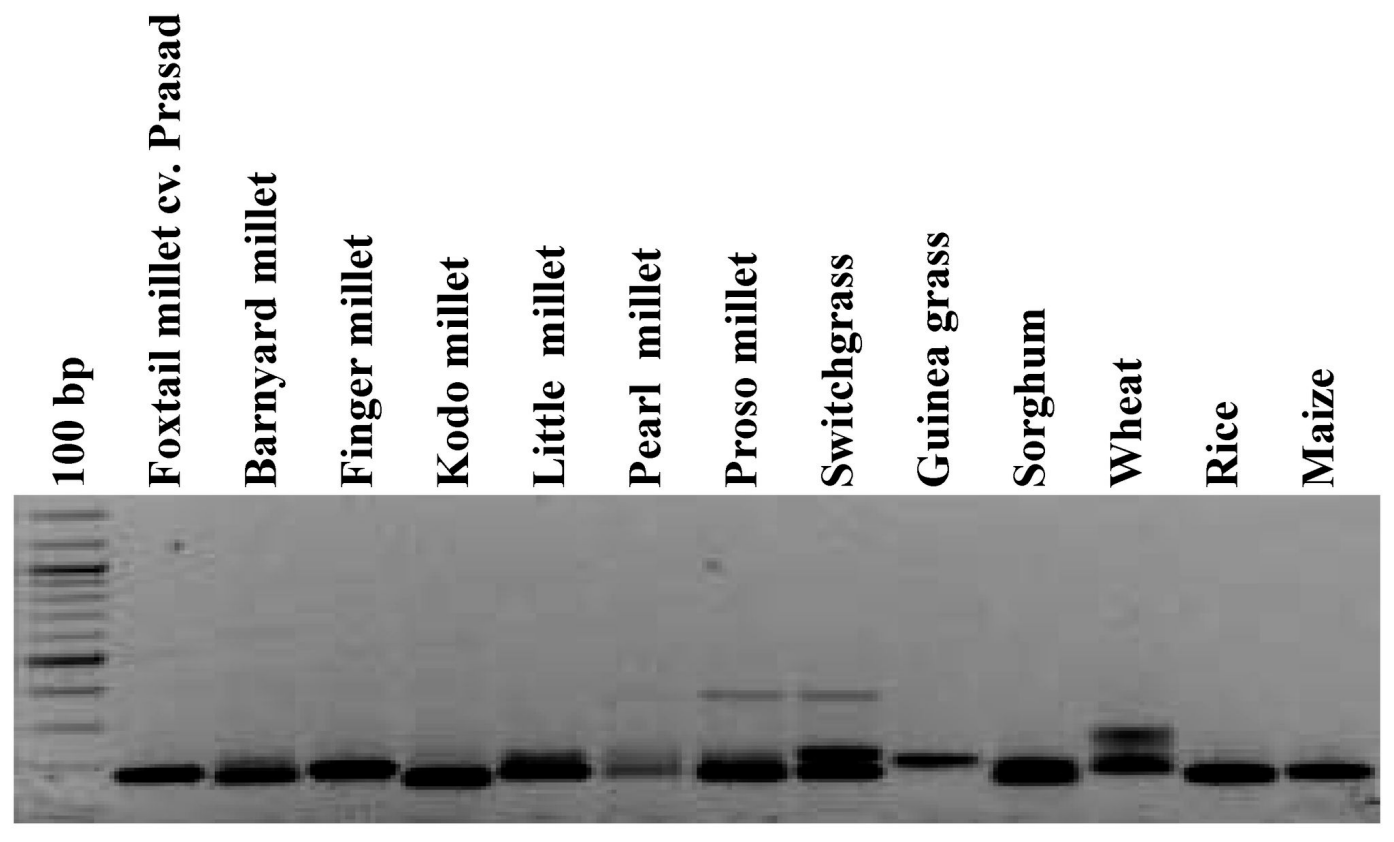
Representative gel showing amplification profiles of microsatellite marker SieSSR226a and its fragment length polymorphism among foxtail millet and related species. The amplicons are resolved in 2% agarose gel along with 100 bp DNA size standard.

In order to investigate the utility of the eSSR markers in cross-genera transferability, the 106 validated set of eSSR markers were used to amplify the genomic DNA of eight millet (barnyard millet, finger millet, kodo millet, little millet, pearl millet, proso millet, switchgrass, guinea grass) and four non-millet species (sorghum, wheat, rice and maize). Of the 106 eSSRs markers assayed, the highest transferability percentage (92.5%) was observed in guinea grass and lowest (80.2%) in finger millet with an average percent transferability of 87.9% (Figure 4, Table 2, Table S4). Markers which showed a consistent amplification profile in other species were scored as being cross-transferable, thus confirms the utility of developed eSSR markers for revealing high cross-genera transferability.

**Table 2:** Percent transferability of 106 eSSR markers in different millet and non-millet species.

S. No	Investigated crop	% transferability
1	Barnyard millet	90.6
2	Finger millet	80.2
3	Kodo millet	87.7
4	Little millet	89.6
5	Pearl millet	88.7
6	Proso millet	88.7
7	Switchgrass	89.6
8	Guinea grass	92.5
9	Sorghum	92.5
10	Wheat	81.1
11	Rice	89.6
12	Maize	84
**Average**		**87.8**

**Figure 5:**
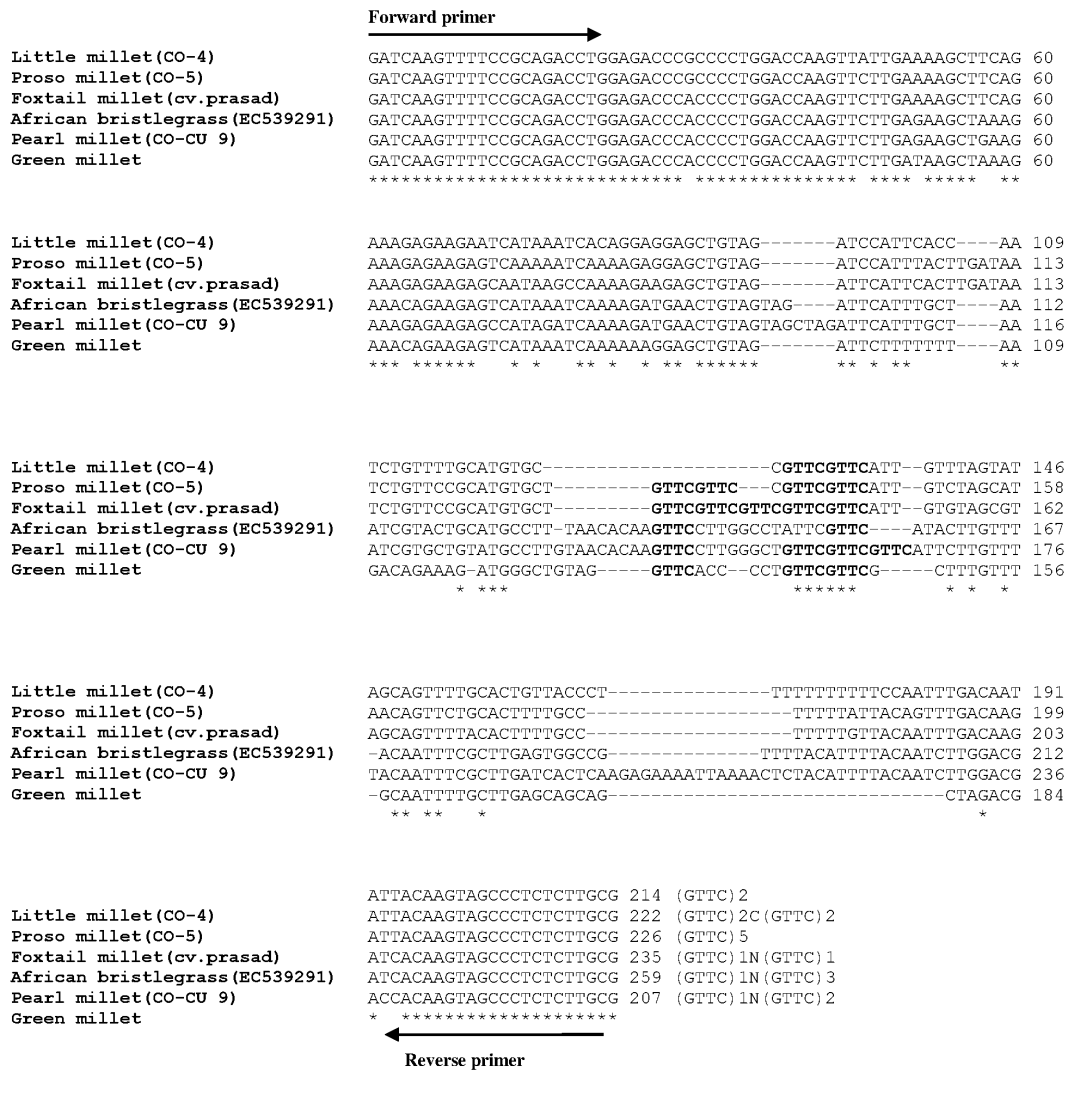
Multiple sequence alignment of SieSSR249 showing the presence of microsatellite repeat motif in millets and its related grass species. Alignment reveals occurrence of variable number of repeat motifs in different species along with multiple point mutations and insertion/deletions.

**Figure 6:**
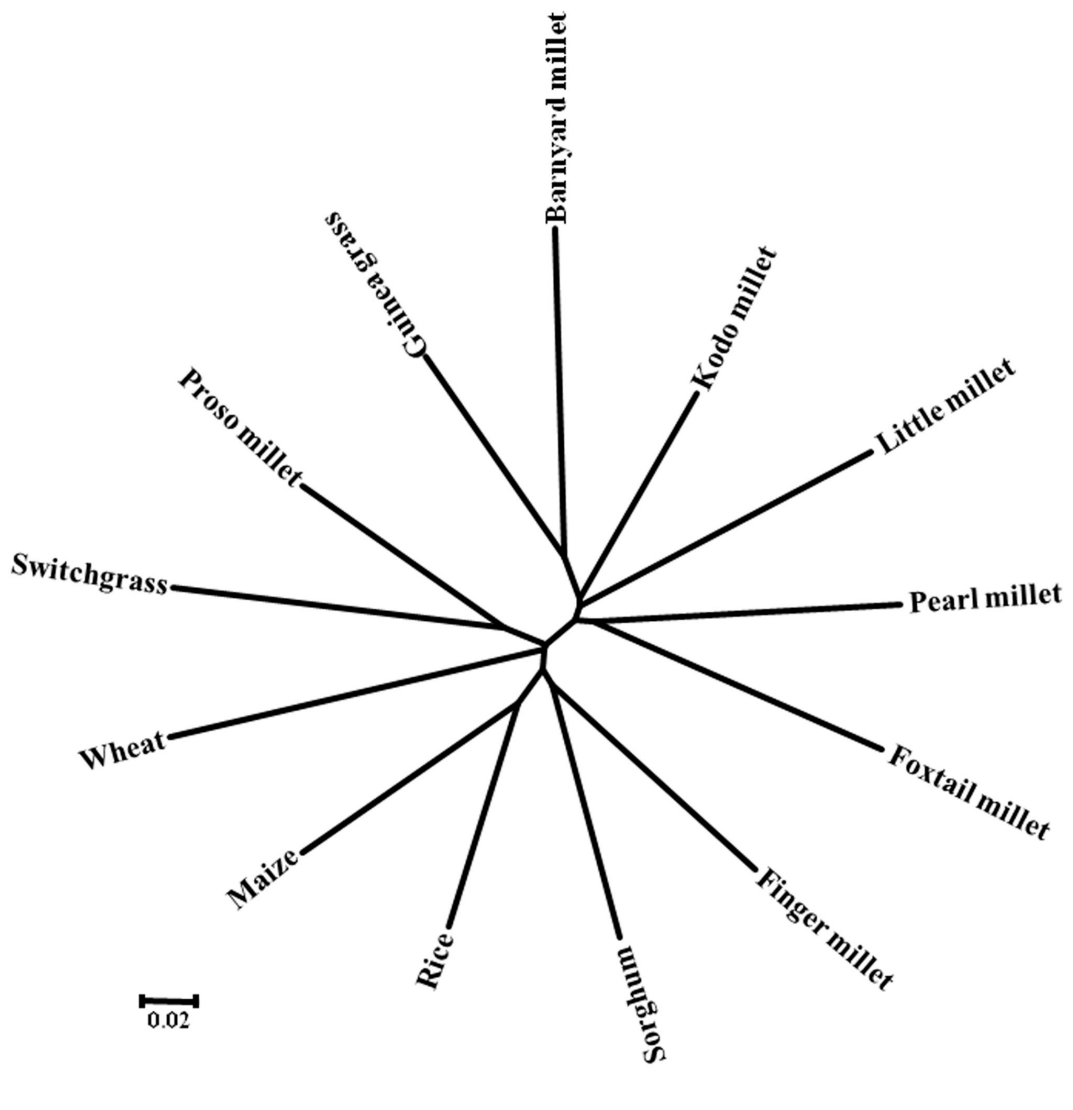
Phylogenetic relationships among thirteen millet and non-millet species using 96 eSSR markers.

Allelic length variations were because of differences in the copy of microsatellite repeats, whereas isolated point mutations in the regions flanking microsatellite may be accountable for polymorphism in the sequence. For instance, the locus SieSSR249 amplified variant alleles from 207 to 259 bp (Figure 5). Sequence analysis for locus SieSSR249 (JK580299) revealed mixed type of allelic distribution with the variable number of repeats in the SSR motifs (GTTC)_n_ accompanied by several point mutations like indels or substitution mutations (Figure 5). Besides, all the 106 microsatellite markers have ability to distinguish the investigated millet and non-millet species belonging to different genera (Figure 6). The variations in the number of alleles per microsatellite marker locus in different species studied is possibly dependent upon ploidy level, nature and number of genotype sets in each species used for analysis.

### Genetic diversity of eSSR markers

**Figure 7:**
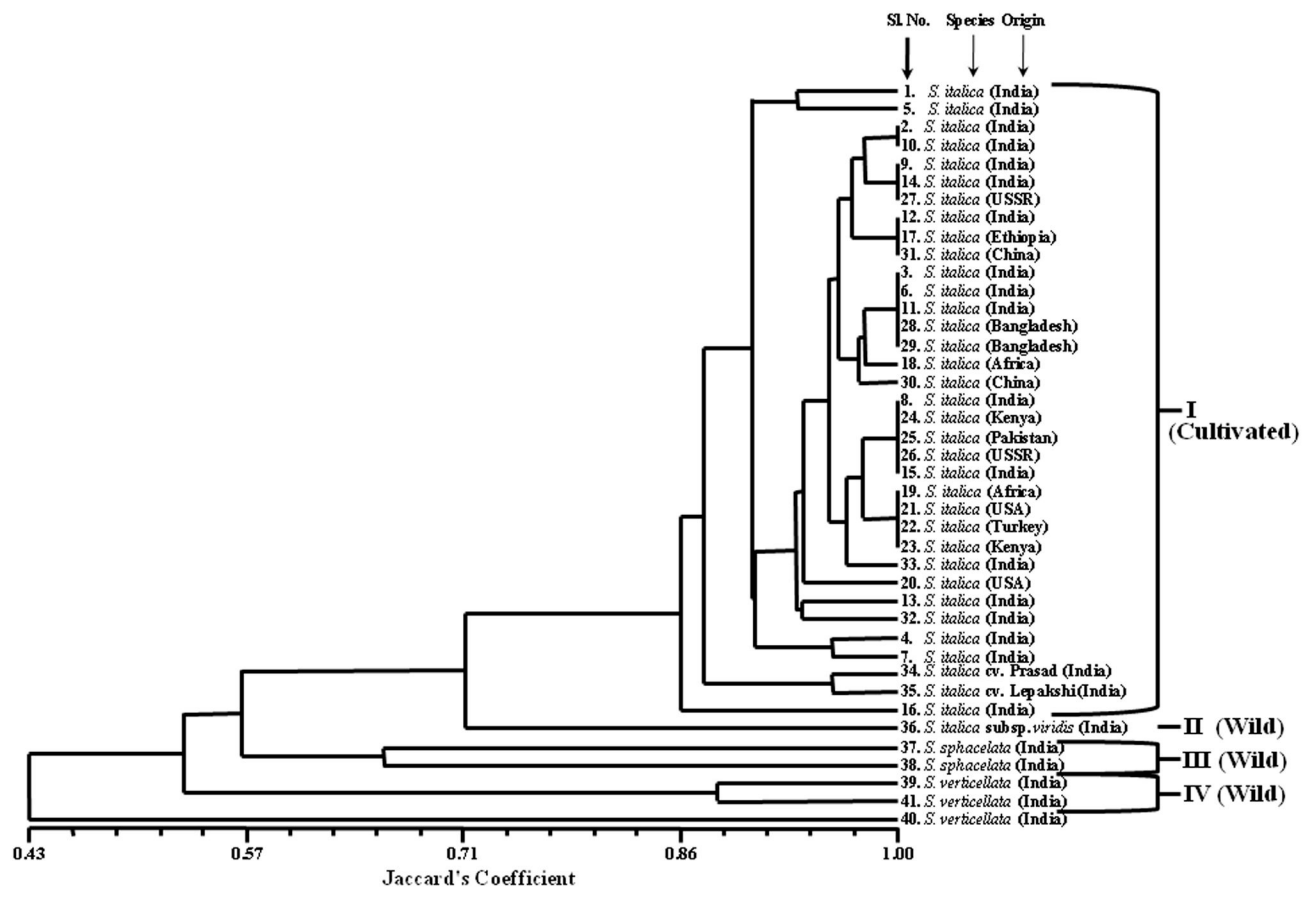
Genetic diversity of 41 *Setaria* species using 40 eSSR markers. Serial numbers of the accessions corresponds to Table S1.

A core set of 35 cultivated *S* *. italica* accessions and 6 related wild species were used to decipher the polymorphic potential of 40 eSSR markers representing the whole genome of foxtail millet (Table S5). In total, 88 alleles were identified with average of 2.2 alleles per locus varying from two to five. The polymorphic information content (PIC) values were extended from 0.000 to 0.48 with a mean of 0.097 (Table S5). The observed heterozygosity (*H*_*O*_) for individual loci ranged from 0.000 to 0.975 with a mean of 0.1. The Nei’s average gene diversity (*Nei*) ranged from 0.000 to 0.608 with a mean of 0.128. Among all the loci analyzed with fixation index (*F*_*IS*_), eighteen loci were found positive representing excess of observed homozygotes whereas seven loci were negative demonstrating heterozygotes with a mean of 0.492 per locus. The Shannon’s Informative Index (*I*) of the loci varied from 0.000 to 1.107 with a mean of 0.248 per locus (Table S5). There was no significant correlation observed between PIC, number of repeat unit and allele number for the 40 markers investigated (data not shown). The level of genetic diversity of 35 cultivated *S* *. italica* accessions and 6 related wild species varied from 0.02 to 0.65 (Table S6). The level of diversity among cultivated *S* *. italica* accessions ranged from 0.02 to 0.23, while among wild species it varied from 0.12 to 0.65. The wild species *S* *. verticillata* (EC539297) showed maximum level of diversity (0.61) with cultivated accessions IC403962 and IC403522A, and minimum diversity (0.24) was obtained between *S* *. italica* sub spp. *viridis* (green millet) and IC403962 (Table S6). The phylogenetic tree constructed in this study using eSSR markers differentiated 35 cultivated *S* *. italica* accessions and 6 related wild species from each other and clustered according to their taxonomic classification. The dendrogram constructed grouped 41 *Setaria* accessions into four distinct clusters, cluster I with 35 accessions comprising cultivated species (foxtail millet, *S* *. italica* ), II with one accession of *S* *. italica* sub sp. *viridis* (green millet), III with two accessions of S. *sphacelata* (African bristle grass) and cluster IV containing two accessions of *S* *. verticillata* (bristly foxtail). One accession of *S* *. verticillata* being diverged from other accessions was grouped separately (Figure 7).

### *In-silico* comparative genome mapping between foxtail millet and other grass species

**Figure 8:**
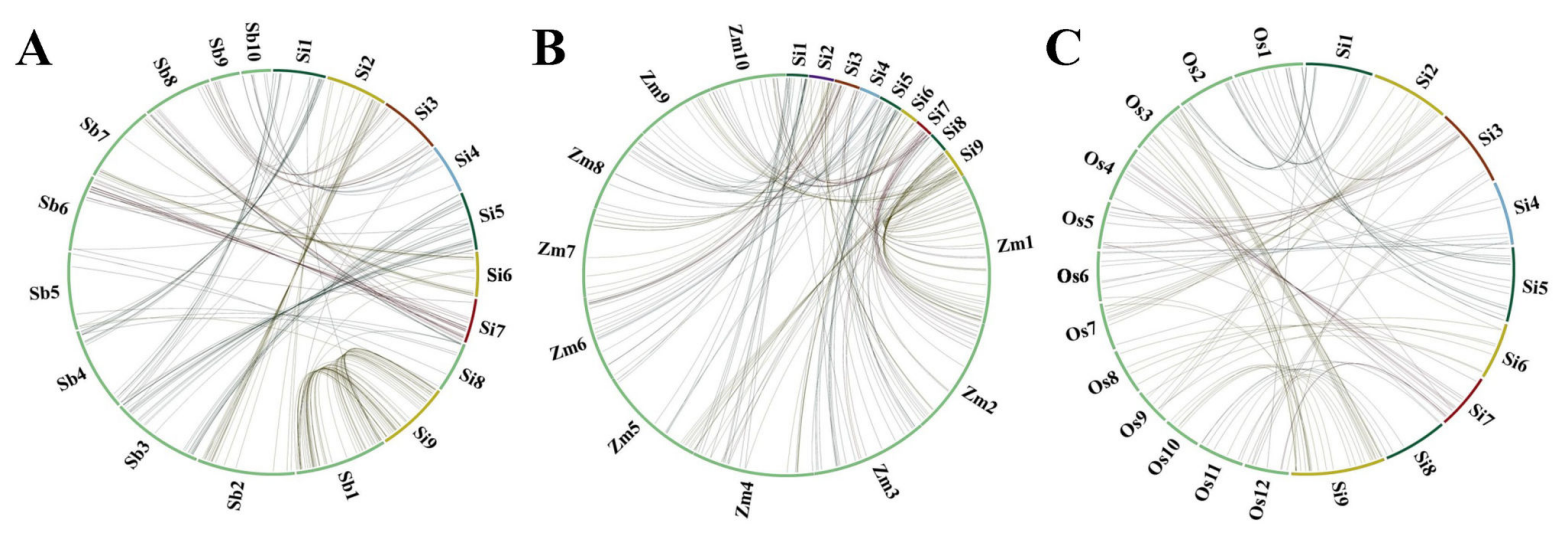
Genome relationships of foxtail millet with other grass species. Comparative mapping between foxtail millet chromosomes with (A) sorghum, (B) maize and (C) rice chromosomes.

The physically mapped 327 eSSR markers on the nine chromosomes of foxtail millet were compared with their physical location on the chromosomes of other related grass genomes of sorghum, maize and rice (Figure 8, Table 3). The comparative genome mapping showed considerably significant proportion of sequence-based orthology and syntenic relationship of eSSR markers distributed over nine foxtail millet chromosomes with sorghum (~68%, 223), maize (~61%, 200) and rice (~42%, 136) chromosomes (Tables S7–S9).

**Table 3:** Summary of microsatellite marker-based comparative mapping showing maximum syntenic relationships of foxtail millet chromosomes with sorghum, maize and rice chromosomes.

**Foxtail millet chromosomes**	**Sorghum chromosomes**	**Maize chromosomes**	**Rice chromosomes**
Chr.1	Chr.4 (17, 68%)	Chr.4 (8, 38.1%) & Chr.5 (6, 28.6%)	Chr.2 (11, 84.6%)
Chr.2	Chr.2 (20, 95.2%)	Chr.7 (10, 52.6%) & Chr.2 (6, 31.6%)	Chr.7 (7, 50%) & Chr.9 (4, 28.6%)
Chr.3	Chr.9 (15, 60%) & Chr.8 (5, 20%)	Chr.6 (10, 50%) & Chr.1 (3, 15%)	Chr. 5 (7, 41.2%) & Chr.12 (3, 17.6%)
Chr.4	Chr.10 (16, 88.9%)	Chr.6 (7, 46.6%) & Chr.9 (5, 33.3%)	Chr.6 (7, 87.5%)
Chr.5	Chr.3 (27, 93.1%)	Chr.3 (14, 53.8%) & Chr.8 (9, 34.6%)	Chr.1 (13, 81.3%)
Chr.6	Chr.7 (12, 75%)	Chr.1 (5, 45.4%) & Chr.4 (4, 36.4%)	Chr.8 (6, 66.7%)
Chr.7	Chr. 6 (15, 57.7%)	Chr.2 (10, 40%) & Chr.10 (9, 36%)	Chr.4 (11, 68.7%)
Chr.8	Chr.5 (4, 40%) & Chr.8 (3, 30%)	Chr.2 (4, 44.4%) & Chr.4 (2, 22.2%)	Chr.1 (4, 44.4%) & Chr.11 (4, 44.4%)
Chr.9	Chr.1 (51, 96.2%)	Chr.1 (29, 53.7%), Chr.9 (10, 18.5%) & Chr5 (9, 16.7%)	Chr.3 (22, 64.7%) & Chr.10 (6, 17.65%)

#### Foxtail millet - sorghum synteny

**Figure 9:**
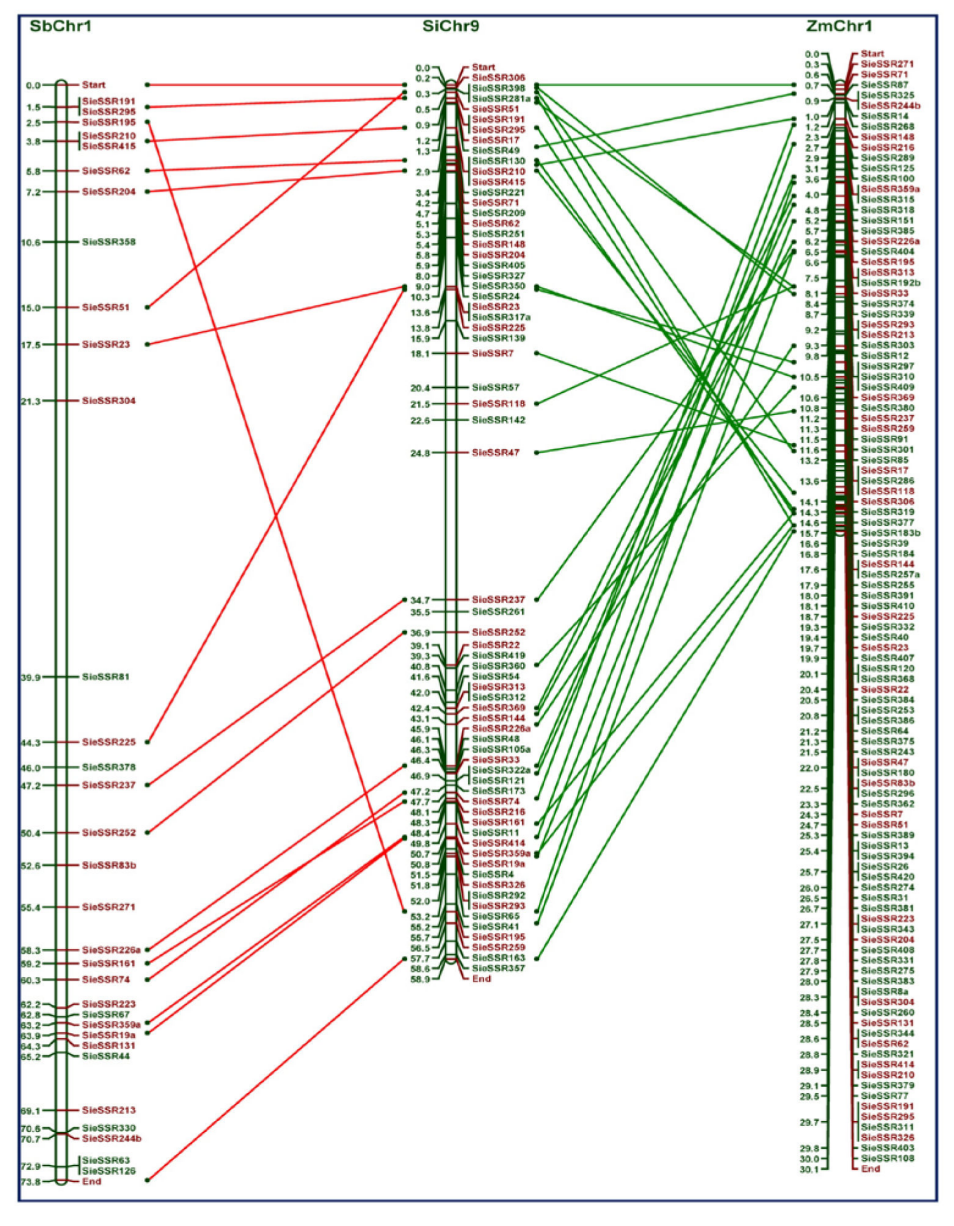
Comparative mapping between foxtail millet chromosome 9 (SiChr9) with sorghum chromosome 1 (SbChr1) and maize (ZmChr1).

The comparative mapping between foxtail millet and sorghum genomes revealed syntenic relationship of 223 eSSR marker loci distributed over nine chromosomes of foxtail millet with 223 genomic regions on 10 chromosomes of sorghum. On an average, ~68% syntenic relationship of microsatellite marker loci between foxtail millet and sorghum chromosomes was observed. The syntenic relationship of eSSR marker loci was maximum between foxtail millet chromosome 9 with sorghum chromosome 1 (96.2%) (Figure 9) followed between foxtail millet chromosome 5 and sorghum chromosome 3 (93.1%), between foxtail millet chromosome 2 and sorghum chromosome 2 (95.2%) and minimum between foxtail millet chromosome 8 and sorghum chromosome 5 (40%) (Figure 8A, Table 3, Table S7).

#### Foxtail millet - maize synteny

Between foxtail millet and maize genomes, 200 eSSR marker loci distributed over nine chromosomes of foxtail millet showed significant matches with 200 genomic regions of ten maize chromosomes. Interestingly, each foxtail millet chromosome showed sytenic relationship with two maize chromosomes thus highlighting the recent whole genome duplication in maize. All the nine foxtail millet chromosomes showed considerable and higher average frequency (~61%) of microsatellite marker-based syntenic relationship with specific maize chromosomes. The physically mapped eSSR markers on the foxtail millet chromosome 5 showed maximum synteny (53.8%) with maize chromosome 3 followed between foxtail millet chromosome 9 and maize chromosome 1 (53.7%) (Figure 9), between foxtail millet chromosome 2 and maize chromosome 7 (52.6%) and minimum between foxtail millet chromosome 1 and maize chromosome 4 (38.1%) (Figure 8B, Table 3, Table S8).

#### Foxtail millet - rice synteny

The microsatellite markers physically mapped on the foxtail millet chromosomes showed least synteny with rice chromosomes with average frequency of ~42% (136 marker loci) which is relatively lower than that with the sorghum and maize chromosomes. Maximum synteny of microsatellite marker loci between foxtail millet chromosome 4 and rice chromosome 6 (87.5%) followed between foxtail millet chromosome 1 and rice chromosome 2 (84.6%), between foxtail millet chromosome 5 and rice chromosome 1 (81.3%) and minimum between foxtail millet chromosome 3 and rice chromosome 5 (41.2%) was observed (Figure 8C, Table 3, Table S9).

**Figure 10:**
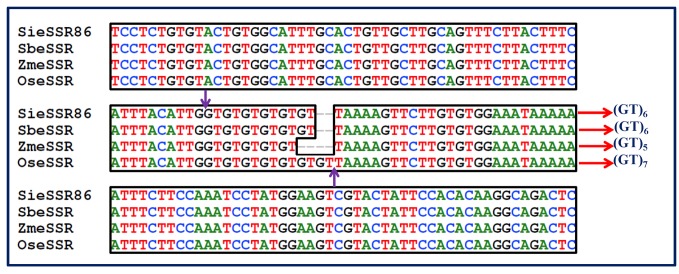
Multiple sequence alignment showing the conservation and expansion/contraction of SSR repeats in the conserved EST sequences encoding for serine–threonine protein kinase disease resistance gene among the chromosomes of foxtail millet (Si), sorghum (Sb), maize (Zm) and rice (Os).

The *in silico* comparative genome mapping between chromosomes of foxtail millet and three other grass family members (maize, sorghum and rice) based on conservation and expansion/contraction of SSR repeats in the EST sequences were analyzed in detail (Figure 10). Interestingly, 27 conserved orthologous set (COS) - eSSR markers that are conserved among the chromosomes of foxtail millet, maize, sorghum and rice were identified. The maximum expansion and contraction of eSSR repeats was observed in 32 (23.5%) of the 223 SSR-carrying EST sequences conserved between foxtail millet and rice, 21 (10.5%) of the 200 EST-SSR sequences conserved between foxtail millet and maize and 14 (6.3%) of the 223 EST-SSR sequences conserved between foxtail millet and sorghum. However, the chromosomes of foxtail millet showing maximum syntenic relationships with maize, sorghum and rice revealed lesser degree of expansion and contraction of eSSR repeats whereas the chromosomes having minimum synteny gave higher degree of expansion and contraction of eSSR repeats. It overall indicated an inverse correlation between variations (expansion/contraction) of SSR repeats in the conserved EST sequences and their syntenic relationships among the chromosomes of four target species under study.

**Figure 11:**
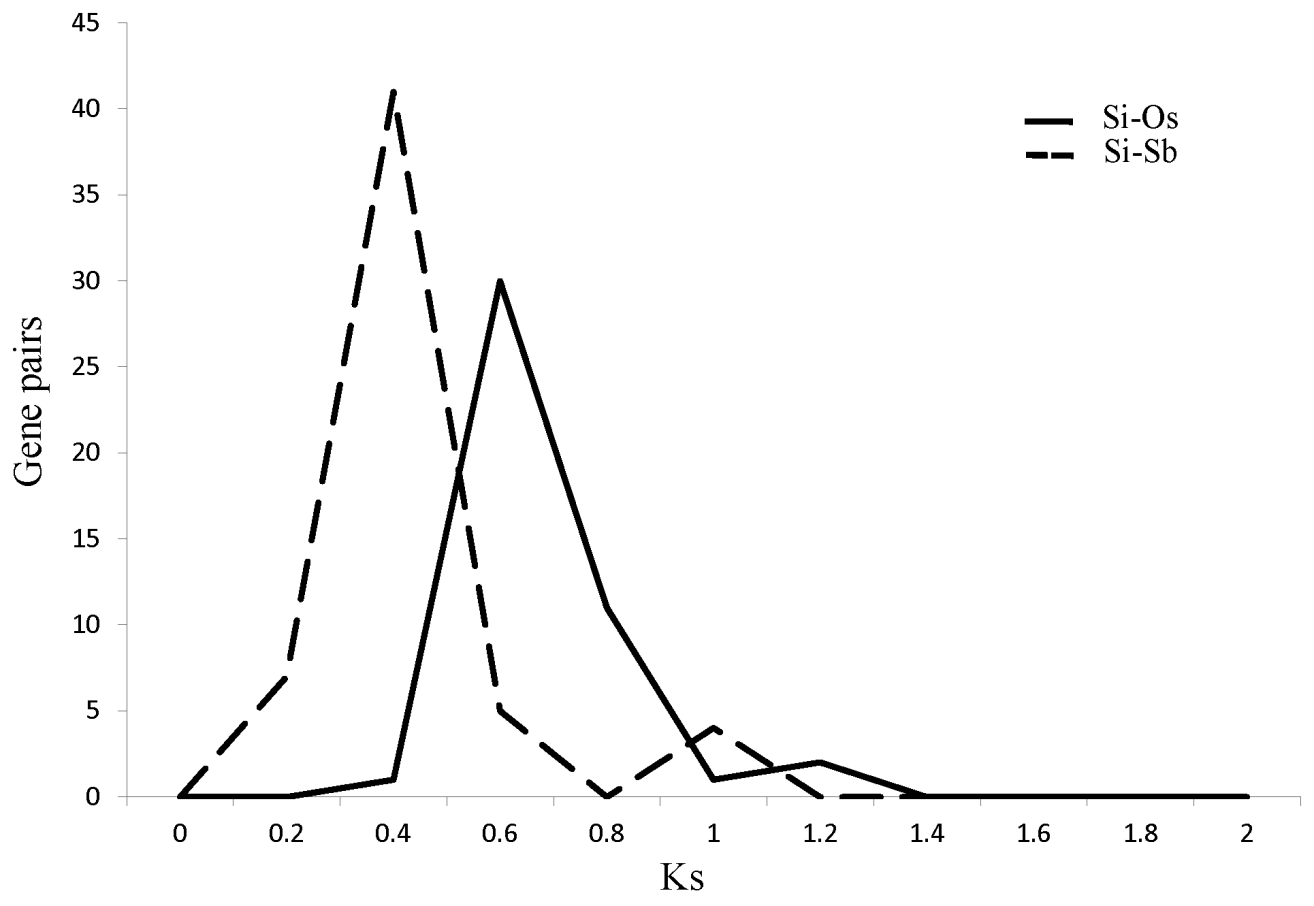
Ks dating of SSR-containing orthologs between foxtail millet (Si) and rice (Os) or sorghum (Sb). Distribution of synonymous substitution per synonymous sites (Ks) (bin size 0.2) of SSR-containing orthologous gene pairs between foxtail millet and rice or sorghum plotted against number of gene pairs.

Synteny analysis of SSR-containing ESTs between foxtail millet and sorghum or rice has resulted in identification of collinear blocks in these genomes and identification of orthologous ESTs. Distribution of synonymous substitution per synonymous site (Ks) with the orthologous gene pairs present in the syntenic blocks were used to determine divergence periods of these crops. Foxtail millet-rice orthologs showed a peak at Ks 0.60-0.65. Assuming a rate of synonymous substitution per synonymous site per year as 6.5 x 10^-9^ for monocots [28], this peak attributes to the period of divergence of the Pooideae and Panicoideae at 46-50 million years ago (mya). The peak at Ks 0.35-0.40 shown by the orthologous pairs of foxtail millet and sorghum dated split of foxtail millet from sorghum at 26-30 mya (Figure 11).

## Discussion

Since the eSSR markers possess significant specificity and high degree of conservation, they are considered to be potential tool for various genotyping applications including studying cross-transferability and phylogenetic relationships and comparative genome mapping in crop species [29-32]. Since the establishment of foxtail millet as a model species for studying the functional genomics of bioenergy grasses, significant progress has been made in the area of development of molecular markers in present days [33]. However, best to our knowledge only one report is available on foxtail millet eSSR markers, where about 26 eSSR markers were developed and its transferability was studied [34]. This scenario motivated us to develop plentiful novel eSSR markers in foxtail millet and demonstrate its usefulness in functional genomics.

The average frequency of ~2% (1/30 kb) non-redundant SSRs in transcribed region of the foxtail millet is within the same range as previously reported for other plant species viz. 1.3% in Tall fescue [35], 1.5% in maize [36], ~2.4% in *Arabidopsis* [37], but low in comparison to barley (3.4%), wheat (3.2%), rice (4.7%), sorghum (3.6%) [36] and alfalfa (3.0%) [38]. The significant variation in the frequency and distribution of eSSRs among different studies is plausibly due to variation in sample sizes, search criteria, size of the data base, tools used for eSSRs development and differences among species studied [11,39].

Among the 534 eSSRs obtained, the tri-nucleotide repeat motifs were the most abundant (321) with a proportion of 60.1%, followed by di- (184, 34.4%), tetra- (21, 3.9%), penta- (5, 0.9%) and hexa- (3, 0.6%) nucleotide repeats. Though foxtail millet eSSRs contained diverse types of repeat motifs, tri-nucleotide repeat motifs were evidenced at higher proportions (both in class I and class II), this observation agrees to the results reported in many crops including cotton [40], barley, wheat, maize, sorghum, rice [36], tall fescue [35], peanut [41] and sesame [42]. The abundance of tri-nucleotide repeat motifs could be explained as expansions or deletions in coding regions can be tolerated more for tri-nucleotide unit repeats, which do not perturb open reading frames [43,44].

Of the tri-nucleotide repeats, AGC/CTG motif was predominant at a percentage of 17.6 followed by AAG/CTT motif at 9%. Each tri-nucleotide motif present in eSSR loci codes a specific amino acid which plays an important role in biological, cellular and metabolic process in plants. In our study, we have found 321 (60%) tri-nucleotides repeats motif representing 14 amino acids including start codon (AUG). The percentage of tri-nucleotide motifs coding for Leucine was highest (~27%) followed by Serine (~20%). A similar observation has been reported in *Catharanthus* *roseus* where also Serine and Leucine were abundant [45].

Primer pairs were effectively designed for 447 (90.3%) sequences in this study, while the remaining 48 (9.7%) sequences could not be used, either due to the presence of very small DNA sequence flanking the SSRs or their unsuitability for designing primers. Of the 447, only 327 eSSRs were physically mapped on the nine chromosomes. The plausible reason for this inability to map the remaining 120 markers is the availability of only ~80% of foxtail millet genome in the public domain, since a draft sequence was published [4].

Out of 447 eSSR markers, 106 were selected for validation where all the eSSR markers produced 100% amplification success rate. An evaluation of the observations pointed that the average number of alleles per locus (2.2) in this study were comparable to previous studies in sesame (2-4 [42]); *Linum* *usitatissimum* (2.26 [46]) and foxtail millet (2.5 [34]), but relatively lower than pigeon pea (4-10 [12]) and sugarcane (7.42 [47]). Of the loci analyzed with fixation index (*F*_*IS*_), eighteen loci were found positive representing excess of observed homozygotes whereas seven loci were negative demonstrating heterozygotes. The mean PIC value in this study was 0.097, which is comparatively lower possibly due to the nature of the markers as EST sequences tend to be highly conserved.

The accomplishment of transferability of eSSR markers rely on the genetic or evolutionary closeness among the species examined. The transferability of foxtail millet SSRs has been studied in twelve different grass species and has revealed different levels of sequence conservations. In this study, the mean percentage (~88%) of successful transferability of the foxtail millet SSRs markers was comparable to a similar result observed by Saha et al. [35], where ~80% of tall fescue eSSR markers showed amplification to *Lolium* species followed by the observation of Varshney et al. [31], where 78.2% of barley markers were conserved in wheat and 75.2% in rye. The high levels of transferability in this study substantiates the applicability of eSSR markers in comparative genome mapping and evolutionary studies in other grass species, especially those lacking sequence information or genetic maps. Sequence analysis of the tested species divulged many point mutations, like single base indels or substitution mutations, along with different repeat number in the SSR motif. Similar observations were also reported in several previous studies [48-50]. The phylogenetic relationships established by 106 eSSR markers among the millet and non-millet were well accordance with the taxonomical classification. For example, species belonging to tribe paniceae were grouped separately from species belong to other tribes.

To assess the applicability of the SSR markers developed, 40 of them were used for analysis of genetic diversity in 41 accessions of *Setaria* . A wider level molecular diversity estimated (0.02-0.65) among 41 accessions suggested the utility of designed genic SSR markers in cultivar identification and genetic diversity studies in foxtail millet. Besides, the developed genic SSR markers have potential for discriminating the 41 accessions from each other and revealing expected phylogenetic relationships based on their taxonomic classification and parentage. Specifically, the genic SSR markers showing higher level of diversity (0.24-0.61) between cultivated and wild species accessions could be utilized in introgression breeding to select suitable inter-specific/genetic hybrids for successful transfer of the genes of agricultural importance like abiotic and biotic stress tolerance from wild species to related cultivated foxtail millet accessions.

Mapping of eSSR markers either genetically or physically on orthologous or syntenic chromosomes of different genomes in related plants was shown in various reports [42,51-56]. Comparative microsatellite marker-based genome mapping revealed higher degree of synteny between foxtail and sorghum (~68%) followed by maize genome (~61%) and rice (~42%). This clearly indicates the declination of synteny with increasing phylogenetic distance among plant species as rice belongs to different subfamily Ehrhartoideae while sorghum and maize share common subfamily Panicoideae with foxtail millet. Such syntenic relationships between foxtail millet and sorghum, maize, rice has also been reported by Gupta et al. [15] and Zhang et al. [42]. This is further evident from the expansion and contraction of SSR repeats in the conserved EST sequences among the chromosomes of foxtail millet, maize, sorghum and rice which resulted in higher evolutionary divergence between foxtail millet and rice and minimum between foxtail millet and sorghum. Despite the fact that our results do not provide a whole genome view due to the limitations of regions studied and biasness of markers used, it highlights the extensive chromosomal rearrangement in the grass chromosomes as evidenced before [5]. Our data (Table 3) suggests colinearity between foxtail millet chromosomes 2, 3 and 9 and rice chromosomes 7 and 9, 5 and 12, 3 and 10, respectively; even at the EST level indicating that these three pairs of rice chromosomes were separately fused to construct three chromosomes of foxtail millet. Among these three fusions, the one that gave rise to foxtail millet chromosome 3 by fusion of rice chromosomes 5 and 12 did not occur in sorghum, indicating that the other two common fusion events occurred before and the fusion of sorghum chromosomes 8 and 9 to form foxtail millet chromosome 3 occurred during or after the divergence of foxtail millet and sorghum. Still, further investigation is mandatory for comprehensively evaluating the level of colinearity between sorghum, maize and rice with foxtail millet genomes.

In summary, the present investigation is the first report on large-scale development and applicability of novel eSSR markers in foxtail millet. Being highly transferable and polymorphic, this novel set of eSSR markers developed here will serve as valuable resource for genetic research in millet and non-millet species on aspects such as comparative mapping, genetic diversity, qualitative and quantitative trait mapping and marker-assisted selection studies.

## Supporting Information

Table S1 Description of plant materials used in the present study. (DOC)

Table S2 Frequency and size distribution of eSSR repeat-motifs mined from 24,828 non-redundant EST sequences of *S* *. italica* . (DOC)

Table S3 Characteristics of the 447 eSSR markers developed in foxtail millet. (XLS)

Table S4 Cross-genera transferability of 106 genic SSRs from foxtail millet to related grass species. (DOC)

Table S5 Summary of genetic diversity of 41 foxtail accessions using 40 eSSR markers. (DOC)

Table S6 Summary of levels of genetic diversity for each *Setaria* accessions. (XLS)

Table S7 Summary of comparative mapping between foxtail millet and sorghum. (DOC)

Table S8 Summary of comparative mapping between foxtail millet and maize using eSSR markers. (DOC)

Table S9 Summary of comparative mapping between foxtail millet and rice using eSSR markers. (DOC)
