# Supplementary material for: Development of eSSR-Markers in Setaria italica and Their Applicability in Studying Genetic Diversity, Cross-Transferability and Comparative Mapping in Millet and Non-Millet Species
Source: PLoS One. 2013 Jun 21;8(6):e67742. doi: 10.1371/journal.pone.0067742 (PMC3689721; doi:10.1371/journal.pone.0067742)
Supplement: Table S1 — (DOC) [file pone.0067742.s001.doc]

**Table S1.** Description of plant materials used in the present study.

| Sl. No. | Common name | Species | Native ID |
| --- | --- | --- | --- |
| 1 | Foxtail millet | *Setaria italica* | IC403579 |
| 2 | Foxtail millet | *S.italica* | IC403846 |
| 3 | Foxtail millet | *S.italica* | IC403962 |
| 4 | Foxtail millet | *S.italica* | IC404133 |
| 5 | Foxtail millet | *S.italica* | IC404144 |
| 6 | Foxtail millet | *S.italica* | IC403483 |
| 7 | Foxtail millet | *S.italica* | IC403522 |
| 8 | Foxtail millet | *S.italica* | IC403522A |
| 9 | Foxtail millet | *S.italica* | IC403717 |
| 10 | Foxtail millet | *S.italica* | IC430739 |
| 11 | Foxtail millet | *S.italica* | IC340216 |
| 12 | Foxtail millet | *S.italica* | IC403899 |
| 13 | Foxtail millet | *S.italica* | IC480201 |
| 14 | Foxtail millet | *S.italica* | IC479674 |
| 15 | Foxtail millet | *S.italica* | IC480117 |
| 16 | Foxtail millet | *S.italica* | IC479243 |
| 17 | Foxtail millet | *S.italica* | GS454 |
| 18 | Foxtail millet | *S.italica* | GS455 |
| 19 | Foxtail millet | *S.italica* | GS456 |
| 20 | Foxtail millet | *S.italica* | GS464 |
| 21 | Foxtail millet | *S.italica* | GS465 |
| 22 | Foxtail millet | *S.italica* | GS493 |
| 23 | Foxtail millet | *S.italica* | GS496 |
| 24 | Foxtail millet | *S.italica* | GS495 |
| 25 | Foxtail millet | *S.italica* | GS1636 |
| 26 | Foxtail millet | *S.italica* | GS1643 |
| 27 | Foxtail millet | *S.italica* | GS1646 |
| 28 | Foxtail millet | *S.italica* | GS1926 |
| 29 | Foxtail millet | *S.italica* | GS1928 |
| 30 | Foxtail millet | *S.italica* | GS2038 |
| 31 | Foxtail millet | *S.italica* | GS1641 |
| 32 | Foxtail millet | *S.italica* | GS450 |
| 33 | Foxtail millet | *S.italica* | GS451 |
| 34 | Foxtail millet | *S.italica* | cv.Prasad |
| 35 | Foxtail millet | *S.italica* | cv.Lepakshi |
| 36 | Green foxtail | *S.italica* subsp.viridis | EC539251 |
| 37 | African bristlegrass | *S. sphacelata* | EC539290 |
| 38 | African bristlegrass | *S. sphacelata* | EC539291 |
| 39 | Bristly foxtail | *S. verticillata* | EC539293 |
| 40 | Bristly foxtail | *S. verticillata* | EC539297 |
| 41 | Bristly foxtail | *S. verticillata* | EC539300 |
| 42 | Barnyard millet | *Echinochloa frumentacea* | CO-LV2 |
| 43 | Finger millet | *Eleusine coracana* | CORA114 |
| 44 | Kodo millet | *Paspalum scrobiculatum* | CO-3 |
| 45 | Little millet | *Panicum sumatrense* | C0-4 |
| 46 | Pearl millet | *Pennisetum glaucum* | CO-CU 9 |
| 47 | Proso millet | *Panicum millaeceum* | CO5 |
| 48 | Switch grass | *Panicum virgatum* | PI421521 |
| 49 | Guinea grass | *Panicum maximum* | SPM92 |
| 50 | Sorghum | *Sorghum bicolor* | CO30 |
| 51 | Wheat | *Triticum aestivum* | PH132 |
| 52 | Rice | *Oryza sativa* | cv. Pusa Basmati |
| 53 | Maize | *Zea mays* | B73 |
