# Supplementary material for: Development of eSSR-Markers in Setaria italica and Their Applicability in Studying Genetic Diversity, Cross-Transferability and Comparative Mapping in Millet and Non-Millet Species
Source: PLoS One. 2013 Jun 21;8(6):e67742. doi: 10.1371/journal.pone.0067742 (PMC3689721; doi:10.1371/journal.pone.0067742)
Supplement: Table S2 — (DOC) [file pone.0067742.s002.doc]

**Table S2.** Frequency and size distribution of eSSR repeat-motifs mined from 24,828 non-redundant EST sequences of *S. italica*.

| **Repeats** | **4** | **5** | **6** | **7** | **8** | **9** | **10** | **11** | **12** | **13** | **14** | **15** | **16** | **17** | **18** | **19** | **20** | **21** | **22** | **23** | **24** | **25** | **26** | **Total (%)** |
| --- | --- | --- | --- | --- | --- | --- | --- | --- | --- | --- | --- | --- | --- | --- | --- | --- | --- | --- | --- | --- | --- | --- | --- | --- |
| AC/GT | 12 | 2 | 32 | 16 | 6 | 3 | 2 | 2 | 1 | 1 | 1 | - | - | - | - | - | - | - | - | - | - | - | - | 78 |
| AG/CT | 4 | - | 23 | 13 | 9 | 9 | 5 | 2 | 2 | 2 | - | 1 | - | - | - | - | - | 1 | - | 1 | - | - | - | 72 |
| AT/AT | 3 | - | 18 | 6 | 1 | 2 | 1 | - | - | - | - | - | - | - | - | - | - | - | - | - | - | - | - | 31 |
| CG/CG | - | - | 3 | - | - | - | - | - | - | - | - | - | - | - | - | - | - | - | - | - | - | - | - | 3 |
| **Total NN** | - | - | - | - | - | - | - | - | - | - | - | - | - | - | - | - | - | - | - | - | - | - | - | **184 (34.45)** |
| AAC/GTT | - | 13 | 8 | 1 | - | 4 | - | - | - | - | - | - | - | - | - | - | - | - | - | - | - | - | - | 26 |
| AAG/CTT | 1 | 31 | 7 | 1 | 5 | 1 | - | - | - | 1 | - | - | - | - | - | - | - | - | - | - | - | - | - | 48 |
| AAT/ATT | - | 3 | - | 1 | - | - | - | - | - | - | - | - | - | - | - | - | - | - | - | - | - | - | - | 4 |
| ACC/GGT | - | 17 | 5 | 3 | 3 | - | - | - | - | - | - | - | - | - | - | - | - | - | - | - | - | - | - | 28 |
| ACG/CGT | - | 15 | 5 | 4 | - | - | - | - | - | - | - | - | - | - | - | - | - | - | - | - | - | - | - | 24 |
| ACT/AGT | - | 9 | 2 | 1 | 1 | - | - | - | - | - | - | - | - | - | - | - | - | - | - | - | - | - | - | 13 |
| AGC/GCT | - | 55 | 28 | 8 | - | 2 | 1 | - | - | - | - | - | - | - | - | - | - | - | - | - | - | - | - | 94 |
| AGG/CCT | - | 13 | 6 | 2 | 1 | - | - | - | - | - | - | - | - | - | - | - | - | - | - | - | - | - | - | 22 |
| ATC/GAT | - | 29 | 2 | 1 | - | - | 2 | - | - | - | - | - | - | - | - | - | - | - | - | - | - | - | - | 34 |
| CCG/CGG | - | 14 | 5 | 3 | 1 | - | - | - | - | - | - | - | - | - | - | - | - | - | - | - | - | - | - | 23 |
| Other NNN | 5 | - | - | - | - | - | - | - | - | - | - | - | - | - | - | - | - | - | - | - | - | - | - | 5 |
| **Total NNN** | - | - | - | - | - | - | - | - | - | - | - | - | - | - | - | - | - | - | - | - | - | - | - | **321 (60.11)** |
| ACAT/ATGT | - | 5 | - | - | - | - | - | - | - | 1 | - | - | - | - | - | - | - | - | - | - | - | - | - | 6 |
| AAAG/CTTT | - | 3 | - | - | - | - | - | - | - | - | - | - | - | - | - | - | - | - | - | - | - | - | - | 3 |
| ATCC/ATGG | - | 2 | - | - | - | - | - | - | - | - | - | - | - | - | - | - | - | - | - | - | - | - | - | 2 |
| ACTC/AGTG | - | - | - | 1 | - | - | - | - | - | - | - | - | - | - | - | - | - | - | - | - | - | - | - | 1 |
| AGAT/ATCT | - | - | - | - | 1 | - | - | - | - | - | - | - | - | - | - | - | - | - | - | - | - | - | - | 1 |
| Other NNNN | - | 8 | - | - | - | - | - | - | - | - | - | - | - | - | - | - | - | - | - | - | - | - | - | 8 |
| **Total NNNN** | - | - | - | - | - | - | - | - | - | - | - | - | - | - | - | - | - | - | - | - | - | - | - | **21 (3.93)** |
| Other NNNNN | - | 5 | - | - | - | - | - | - | - | - | - | - | - | - | - | - | - | - | - | - | - | - | - | **5 (0.93)** |
| AAGCGC/CGCTTG | - | - | 2 | - | - | - | - | - | - | - | - | - | - | - | - | - | - | - | - | - | - | - | - | 2 |
| Other NNNNNN | - | 1 | - | - | - | - | - | - | - | - | - | - | - | - | - | - | - | - | - | - | - | - | - | 1 |
| **Total NNNNNN** | - | - | - | - | - | - | - | - | - | - | - | - | - | - | - | - | - | - | - | - | - | - | - | **3 (0.56)** |
| **Total eSSRs** | - | - | - | - | - | - | - | - | - | - | - | - | - | - | - | - | - | - | - | - | - | - | - | **534** |
