# Supplementary material for: Development of eSSR-Markers in Setaria italica and Their Applicability in Studying Genetic Diversity, Cross-Transferability and Comparative Mapping in Millet and Non-Millet Species
Source: PLoS One. 2013 Jun 21;8(6):e67742. doi: 10.1371/journal.pone.0067742 (PMC3689721; doi:10.1371/journal.pone.0067742)
Supplement: Table S4 — (DOC) [file pone.0067742.s004.doc]

**Table S4.** Cross-genera transferability of 106 genic SSRs from foxtail millet to related grass species.

| **Sl.No.** | **Primer ID** | **No. of alleles** | **Obtained Alleles** | | | | | | | | | | | | | **Transferability %** |
| --- | --- | --- | --- | --- | --- | --- | --- | --- | --- | --- | --- | --- | --- | --- | --- | --- |
|  |  | **(Size range of alleles in bp)** | **Foxtail millet** | **Barnyard millet** | **Finger millet** | **Kodo millet** | **Little millet** | **Pearl millet** | **Proso millet** | **Switchgrass** | **Guinea grass** | **Sorghum** | **wheat** | **Rice** | **Maize** |  |
| 1 | SieSSR3 | 9(165-750) | 180 | 165 | 250 | 180 | 180 | 165, 300 | 360 | 260 | 750 | 600 | 260 | 200 | 200 | 100 |
| 2 | SieSSR12 | 7(151-550) | 250 | 150 | NA | 250 | 220, 300 | 320 | NA | 150 | 250, 550 | NA | NA | 250 | 150, 180 | 67 |
| 3 | SieSSR13 | 2(166-450) | 166 | 166 | 166 | 166 | 166 | 166 | 166 | 166 | 166 | 166, 450 | 166 | 166 | 166 | 100 |
| 4 | SieSSR14 | 3(237-500) | 237 | 237, 500 | 237 | 237 | 237 | 237 | 237 | 237 | 237, 500 | 237 | 260, 500 | 237 | 237 | 100 |
| 5 | SieSSR15 | 3(125-236) | 236 | 227, 125 | 236 | 236 | 236 | 236 | 236 | NA | 236 | 236 | 236 | 236 | 236 | 92 |
| 6 | SieSSR17 | 1(110) | 100 | 110 | 110 | 110 | 110 | 110 | 110 | 110 | 110 | 110 | 110 | 110 | 110 | 100 |
| 7 | SieSSR18 | 1(236) | 236 | 236 | 236 | 236 | 236 | 236 | 236 | 236 | 236 | 236 | 236 | 236 | 236 | 100 |
| 8 | SieSSR19c | 1(232) | 232 | 232 | 232 | NA | NA | NA | NA | 232 | NA | NA | NA | NA | NA | 25 |
| 9 | SieSSR20 | 1(234) | 234 | 245 | 234 | 234 | 234 | 234 | 234 | 234 | 234 | 234 | 234 | 234 | 234 | 100 |
| 10 | SieSSR21 | 2(104-300) | 104 | 104 | 104 | 104 | 104 | 104 | 104 | 300 | 104 | 104 | 104 | 104 | 104 | 100 |
| 11 | SieSSR22 | 3(275-300) | 289 | 275 | 275 | 300 | 289 | 289 | 289 | 275 | 289 | 289 | 300 | 289 | 289 | 100 |
| 12 | SieSSR23 | 2(397-407) | 407 | 407 | 407 | 407 | 407 | 407 | 397 | 407 | 397 | 407 | 407 | 407 | 407 | 100 |
| 13 | SieSSR24 | 4(210-270) | 249 | NA | 270 | 270 | 249 | 220 | 270 | 210 | 270 | 270 | NA | NA | 220 | 75 |
| 14 | SieSSR25 | 2(249-500) | 249 | 249 | 249 | 249 | 249 | 249 | 249 | 249 | 249 | 249 | 550 | 249 | 249 | 100 |
| 15 | SieSSR26 | 5(162-380) | 162, 320 | 162, 190 | 162 | 162 | 162 | 162 | 162 | NA | 190 | 162 | 225, 380 | 162, 320 | 162, 190 | 92 |
| 16 | SieSSR27 | 1(166) | 166 | 166 | 166 | 166 | 166 | 166 | 166 | 166 | 166 | 166 | 166 | 166 | 166 | 100 |
| 17 | SieSSR28 | 1(102) | 102 | 102 | 102 | 102 | 102 | 102 | 102 | 102 | 102 | 102 | 102 | 102 | 102 | 100 |
| 18 | SieSSR29 | 1(166) | 166 | 166 | 166 | 166 | 166 | 166 | 166 | 166 | 166 | 166 | 166 | 166 | 166 | 100 |
| 19 | SieSSR30 | 2(294-305) | 294 | 305 | 294 | 294 | 294 | 305 | 294 | 305 | 294 | 294 | 294 | 294 | 294 | 100 |
| 20 | SieSSR31 | 4(210-320) | 217 | 225, 320 | 217 | 217 | 217 | 210 | 217 | 225 | 217 | 217 | NA | 217 | 217 | 92 |
| 21 | SieSSR33 | 3(275-1200) | 275 | 275 | 275 | 275 | 275 | 275 | 275 | 800, 1200 | 275 | 275 | 275 | 275 | 275 | 100 |
| 22 | SieSSR34 | 2(206-275) | 206 | 206 | 206 | 206 | 206, 275 | 206 | 206 | 206 | 206 | 206 | 206 | 206 | 206 | 100 |
| 23 | SieSSR35 | 4(180-310) | 225 | 180, 225 | 225, 250 | 225 | 225, 310 | 225 | 180, 225, 250 | 225 | 180, 225, 310 | 225 | 225 | 180, 225 | 225 | 100 |
| 24 | SieSSR36 | 1(162) | 162 | 162 | 162 | 162 | 162 | 162 | 162 | 162 | 162 | 162 | 162 | 162 | 162 | 100 |
| 25 | SieSSR37 | 3(165-220) | 187, 220 | 187 | 187 | 187 | 187 | 187 | 165 | 187 | 187 | 187 | 187 | 187 | 187 | 100 |
| 26 | SieSSR38 | 4(185-450) | 200 | 200 | 450 | 200 | 200 | 200 | 200 | 200 | 200 | 200, 340 | 185 | 200 | 200 | 100 |
| 27 | SieSSR39 | 4(223-375) | 223 | 223, 375 | 223, 375 | 223 | 223 | 223, 250 | 223, 250 | 223 | 223, 310 | 223 | 223 | 223, 375 | 223 | 100 |
| 28 | SieSSR40 | 2(198-285) | 198 | 198 | 198 | 198 | 198 | 198 | 198, 285 | 198 | 198, 285 | 198 | 198 | 198 | 198 | 100 |
| 29 | SieSSR41 | 1(119) | 119 | 119 | 119 | 119 | 119 | 119 | 119 | 119 | 119 | 119 | 119 | 119 | 119 | 100 |
| 30 | SieSSR42 | 1(191) | 191 | NA | NA | NA | 191 | 191 | 191 | NA | NA | 191 | NA | 191 | 191 | 50 |
| 31 | SieSSR43 | 7(150-400) | 300 | 320 | 320 | 400 | 350 | 230 | 150 | 230 | 230 | 260 | 230 | 230 | 300 | 100 |
| 32 | SieSSR44 | 9(140-700) | 240, 600 | 140, 200 | 700 | 290 | 400 | 600 | 300 | 500 | 500 | 400 | 240 | 240 | 240 | 100 |
| 33 | SieSSR61 | 2(100-120) | 100 | 100 | 120 | 100 | 100 | 100 | 100 | 100 | 100 | 100 | 100 | 100 | 100 | 100 |
| 34 | SieSSR65 | 5(180-280) | 254 | 180 | 254 | 254 | 254 | 254 | 254 | 265 | 280 | 220 | 180 | 220 | 220 | 100 |
| 35 | SieSSR79 | 1 (132) | 132 | NA | NA | NA | NA | NA | NA | NA | NA | NA | NA | NA | NA | 0 |
| 36 | SieSSR84 | 7(180-800) | 200 | 280 | NA | 800 | 200, 500 | NA | 180 | 180 | 180 | 180 | 180, 500 | NA | NA | 67 |
| 37 | SieSSR89 | 5(150-300) | 269 | 150 | NA | NA | 269 | 220 | 200, 269 | 200, 269 | 269 | NA | 200, 300 | 250 | 200 | 75 |
| 38 | SieSSR101a | 5(164-360) | 164 | 190 | 190 | 164 | 164, 210 | 200 | 164 | 190 | 174 | 174 | 174 | 174 | 174 | 100 |
| 39 | SieSSR101b | 8(230-700) | 300 | 230, 600 | 500, 700 | 500 | 230, 350 | 200 | 200, 350 | 230, 260 | 260, 700 | 260, 350 | 230, 350 | 260, 500 | 450 | 100 |
| 40 | SieSSR112 | 4(248-300) | 160, 248 | NA | NA | NA | NA | NA | NA | 230 | 248 | 300 | 300 | NA | NA | 33 |
| 41 | SieSSR113 | 3(207-350) | 207 | 350 | 350 | 207 | 207 | 207 | 207 | 207 | 207 | 305 | NA | 305 | 350 | 92 |
| 42 | SieSSR114 | 9(170-600) | 600, 450 | 170 | 650 | 260 | 360 | 260 | 280 | 280 | 360, 450 | 450 | 450 | 290, 450 | 400 | 100 |
| 43 | SieSSR122 | 6(141-500) | 250, 300, 500 | 290 | 290 | 290 | 320 | 300 | 200 | 300 | 300 | 320 | 300 | 320 | 320 | 100 |
| 44 | SieSSR142 | 2(370-550) | 370 | 370 | 370 | 370 | 370 | 370 | 370 | 370, 550 | 370 | 370 | 370 | 370 | 370 | 100 |
| 45 | SieSSR143 | 5(230-500) | 490 | 250, 470 | NA | 470 | 250, 500 | 470 | 490 | 250 | 470 | 250 | NA | NA | NA | 67 |
| 46 | SieSSR145 | 7(140-600) | 140, 210 | NA | NA | 160 | 160 | 600 | 160, 300 | 200 | 140, 200 | 300 | NA | NA | NA | 58 |
| 47 | SieSSR154 | 5(210-410) | 254 | 254 | NA | NA | NA | 254 | 220 | 210, 410 | 350 | NA | 310 | 400 | NA | 58 |
| 48 | SieSSR156 | 6(180-800) | 180 | NA | 180, 230 | 280 | NA | 520 | NA | NA | 550 | 550 | NA | 800 | 800 | 58 |
| 49 | SieSSR159 | 5(231-510) | 231 | 239 | 239 | 250 | 250 | 250 | 510 | 239, 400 | 239 | 239 | 450 | 250 | 250 | 100 |
| 50 | SieSSR161 | 7(200-750) | 310 | 200, 280 | NA | NA | 280 | 310 | 310 | 310 | 310 | 310, 200 | 600, 750 | 310 | 550 | 83 |
| 51 | SieSSR164 | 7(150-650) | 150 | 180 | 230, 300 | 180 | 150, 220 | 200 | 230, 300 | 180 | 150, 650 | 400, 650 | 300 | 300, 400 | 200, 150 | 100 |
| 52 | SieSSR170 | 7(160-450) | 250, 350 | 200 | 250 | 220 | 250 | 160, 220, 450 | 180, 220 | 200 | 180 | 450 | 220 | 200, 420 | 277 | 100 |
| 53 | SieSSR173 | 4(120-290) | 280 | 300 | NA | NA | 120 | 260 | NA | 350 | 260 | 150 | 120, 260 | 280 | NA | 67 |
| 54 | SieSSR175 | 7(110-510) | 239 | 110, 310 | 340 | 340 | 110 | NA | 110 | 110 | 340 | NA | NA | 510 | NA | 67 |
| 55 | SieSSR179b | 8(170-465) | 450 | 400 | 330, 465 | 465 | 250 | 250 | 465 | 210 | 400 | 300 | 170 | 465 | 300 | 100 |
| 71 | SieSSR183a | 7(140-300) | 218 | 230 | 245 | 230 | 200, 245 | 200 | 140 | 200 | 280 | 300 | 140 | 280 | 160, 245 | 100 |
| 57 | SieSSR193 | 2(247-300) | 247 | 247 | 247 | 247 | 300 | NA | NA | NA | 247 | 247 | 247 | 247 | 247 | 75 |
| 58 | SieSSR199 | 7(135-620) | 144 | 160 | 200 | 144 | 144 | 135 | 135 | 152 | NA | 600 | 620 | 180 | NA | 83 |
| 59 | SieSSR208 | 5(150-410) | 410 | 250 | NA | NA | 230 | 230 | 230, 360 | 230 | 230 | NA | NA | NA | 150 | 58 |
| 60 | SieSSR211 | 8(110-600) | 230, 600 | 125, 230 | 125 | 140, 230 | 125, 350 | 110, 230 | 125 | 140, 450 | 800 | 140 | 230 | 140, 230 | 140 | 100 |
| 61 | SieSSR212 | 4(180-280) | 256 | 256 | 256 | 280 | 256 | 256 | 256, 280 | 256 | 280 | 180 | 256 | 256, 280 | 200 | 100 |
| 62 | SieSSR213 | 2(211-223) | 223 | 223 | 223 | 211 | 223 | 223 | 223 | 223 | 223 | 211 | 223 | 223 | 223 | 100 |
| 63 | SieSSR216 | 7(200-900) | 700 | 700 | 200 | 257 | 720 | 700 | 700 | 700 | 680 | 900 | 700 | NA | NA | 83 |
| 64 | SieSSR225 | 3(160-600) | 160 | NA | NA | NA | NA | NA | NA | 230 | NA | 160 | 600 | 600 | 600 | 42 |
| 65 | SieSSR226a | 8(130-480) | 140 | 130, 380 | 130, 380, 400 | 130, 400 | 190, 270, 400 | 240, 310 | 310 | 310 | 400, 480 | 130, 480 | 310 | 130, 400 | 480 | 100 |
| 66 | SieSSR235 | 8(155-460) | 182, 380 | 410, 460 | 200 | 155 | 182, 155 | NA | 180 | NA | 320, 410 | 320 | 320 | 320 | 460 | 83 |
| 67 | SieSSR236 | 3(297-325) | 297 | 297 | NA | NA | NA | 297 | 325 | NA | NA | 297 | 297 | 297 | 320 | 58 |
| 68 | SieSSR240 | 6(160-500) | 160 | 200, 210 | NA | 236 | 210 | NA | 230 | 240 | NA | 230 | NA | NA | 500 | 58 |
| 69 | SieSSR243 | 10(105-380) | 191 | 160, 200 | 360 | 145, 380 | 270, 380 | 270, 380 | 160, 360 | 145, 160 | 145, 180 | 145, 180 | 105, 360 | 120 | 105 | 100 |
| 70 | SieSSR247 | 5(110-220) | 122 | 122 | 220 | 195 | 122 | 205 | 205 | 205 | 205 | 205 | 205 | 205 | 110 | 100 |
| 71 | SieSSR249 | 6(207-259) | 235 | 214 | 235 | 222 | 214 | 207, 235, 259 | 226 | 222 | 222 | 235 | 235 | 235 | 235 | 100 |
| 72 | SieSSR251 | 9(110-800) | 110, 200, 400 | 110, 800 | NA | 120 | 200, 250 | NA | 270 | 270 | 310 | 150 | NA | 800 | 150 | 75 |
| 73 | SieSSR262 | 5(107-260) | 107 | 107, 160 | 107, 160 | 107 | 107 | 210 | 180 | 180 | 210 | 160 | 107, 260 | 210 | 260 | 100 |
| 74 | SieSSR271 | 8(120-400) | 210 | 120, 400 | 300 | 350, 400 | 400 | 350 | 230 | 230, 320 | 350 | NA | 230, 160 | 210 | 210 | 92 |
| 75 | SieSSR279 | 9(120-650) | 230 | 200, 300 | 280 | 160, 200 | 200 | 650 | 280, 400 | 280, 400 | 300 | 120 | 320 | 300 | 400 | 100 |
| 76 | SieSSR281a | 4(140-300) | 140 | 180 | NA | 240 | 140 | 140 | 180 | 180 | 180 | 240 | 240 | 180, 240 | 240 | 92 |
| 77 | SieSSR283 | 7(240-600) | 300 | 300, 400 | 240 | 500 | 240, 320 | NA | 340 | 360 | 600 | 410 | 600 | 500 | NA | 83 |
| 78 | SieSSR292 | 1(149) | 149 | 149 | 149 | 149 | 149 | 149 | 149 | 149 | 149 | 149 | 149 | 149 | 149 | 100 |
| 79 | SieSSR302 | 2(254-270) | 254 | 254 | 270 | 254 | 270 | 254 | 270 | 270 | 270 | 270 | 270 | 254 | 270 | 100 |
| 80 | SieSSR 322b | 6(150-400) | 242 | 242, 400 | 242 | 242 | 400 | 350 | 300 | 400 | 242 | 150, 270 | 150 | 300 | 150, 300 | 100 |
| 81 | SieSSR323 | 7(140-350) | 171 | 190, 300 | 171 | 190 | 171 | 140 | 171 | 140 | 171 | 270 | 300 | 200, 350 | 171 | 100 |
| 82 | SieSSR332 | 4(121-400) | 121 | NA | NA | 220 | 220 | NA | NA | NA | 220 | 250 | NA | 250, 300 | 300 | 50 |
| 83 | SieSSR333 | 7(120-700) | 280 | NA | 180 | 202 | 180, 280 | 280 | 300 | 325 | 200 | 700 | 120 | 120 | 120 | 92 |
| 84 | SieSSR345 | 4(139-300) | 139 | 210 | 210 | 210 | 210 | 210 | 210 | 139 | 139, 220 | 215 | 300 | 220 | 220 | 100 |
| 85 | SieSSR349 | 6(150-500) | 242 | 242 | 500 | 242 | 300 | 242 | 255 | 255 | 255 | 200 | 242, 300 | 150 | 200 | 100 |
| 86 | SieSSR350 | 5(220-450) | 300 | 300 | 300 | 300 | 300 | 240, 300 | 300, 450 | 300 | 300 | 220 | 300 | 220, 400 | 240 | 100 |
| 87 | SieSSR352 | 3(128-350) | 128 | 128 | 128 | 128 | 128 | 128 | 128 | 150 | 128, 300 | 128 | 128, 350 | 128 | 150 | 100 |
| 88 | SieSSR363 | 6(250-420) | 410 | 250, 420 | 410 | 410 | 250, 410 | 410 | NA | 280, 300, 410 | 300 | 300, 420 | NA | 420 | NA | 75 |
| 89 | SieSSR368 | 7(160-500) | 160 | 350 | 500 | 240 | 220, 260, 350 | 260, 420 | 260, 420 | 500 | 220, 420 | 260, 160 | 350, 500 | 260 | 350, 420 | 100 |
| 90 | SieSSR371 | 3(122-145) | 122 | 135 | NA | NA | 145 | 122 | 122 | 122 | 122 | 122 | 122 | 122 | 122 | 92 |
| 91 | SieSSR373 | 5(107-400) | 107 | 107 | 107 | 107 | 107 | 107 | 107, 400 | 107 | 107, 200, 400 | 107 | 107, 360 | 107, 260 | 107 | 100 |
| 92 | SieSSR382 | 8(160-650) | 350 | 470 | 350 | 160, 250 | 250 | 650 | 180 | 160 | 350, 470 | 200 | 160 | 180 | 180 | 100 |
| 93 | SieSSR384 | 5(130-280) | 183 | 130 | 200, 280 | 280 | 180 | 160 | 160 | 160 | 160 | 160 | 160 | 130 | 130 | 100 |
| 94 | SieSSR401 | 7(190-350) | 236 | 190 | NA | 236 | NA | NA | NA | 225, 310 | 190 | 300 | 250 | 200, 230 | 200, 310 | 67 |
| 95 | SieSSR402 | 9(150-600) | 158 | 250 | 250 | 150 | 300, 390 | 410 | 200 | 500 | NA | 390 | NA | 600 | NA | 75 |
| 96 | SieSSR411 | 5(220-680) | 450 | 680 | NA | 500, 680 | NA | 500 | 220 | 220, 500, 680 | 500, 680 | 250, 680 | 600 | 600 | 680 | 83 |
| 97 | SieSSR91 | 4 (100-300) | 132 | 100 | 100 | 132 | 132 | 132 | 132 | 132 | 132, 210 | 132 | 100, 132 | 300 | 100, 132 | 100 |
| 98 | SieSSR390 | 4 (140-400) | 140 | 140 | 350 | 300 | 350, 400 | 400 | 350 | 300 | 400 | 300 | 300 | 140, 300 | 140 | 100 |
| 99 | SieSSR100 | 3 (110-150) | 120 | 120 | 110 | 110 | 120 | 110 | 120 | 120 | 150 | 120 | 120 | 120 | 110 | 100 |
| 100 | SieSSR165 | 3 (130-650) | 650 | NA | 130 | NA | NA | 220 | 130, 650 | 130 | 650 | 130 | 130 | NA | NA | 66.8 |
| 101 | SieSSR272 | 5 (260-400) | 277 | 300 | 300 | 277 | NA | 400 | 277 | NA | 260, 310 | 277 | NA | 260 | 277 | 75 |
| 102 | SieSSR336 | 3 (115-169) | 169 | 169 | 169 | 169 | 169 | 169 | 130, 169 | 130 | 115, 169 | 169 | NA | 169 | 169 | 91.66667 |
| 103 | SieSSR403 | 5 (110-300) | 279 | 279 | 300 | 225 | 279 | 110 | 140 | NA | 110, 279 | 140 | NA | 110 | NA | 75 |
| 104 | SieSSR407 | 4 (130-200) | 176 | 200 | 200 | 200 | 176 | 160 | 130, 176 | 130 | 160 | 176 | 200 | 176, 200 | 176 | 100 |
| 105 | SieSSR274 | 3 (150-240) | 222 | 150 | 222, 240 | 222 | 222 | 222 | 222 | 222 | 222 | 222 | 150, 222 | 222 | 222 | 100 |
| 106 | SieSSR120 | 3 (110-210) | 210 | 210 | 110 | 170 | 210 | 110 | NA | 110 | 110, 210 | 110 | 110 | 210 | NA | 83.33333 |
|  |  |  |  | **90.6 %** | **80.2 %** | **87.7 %** | **89.6 %** | **88.7 %** | **88.7 %** | **89.6 %** | **92.5 %** | **92.5 %** | **81.1 %** | **89.6 %** | **84 %** | **Avg = 87.8%** |
