# Supplementary material for: Development of eSSR-Markers in Setaria italica and Their Applicability in Studying Genetic Diversity, Cross-Transferability and Comparative Mapping in Millet and Non-Millet Species
Source: PLoS One. 2013 Jun 21;8(6):e67742. doi: 10.1371/journal.pone.0067742 (PMC3689721; doi:10.1371/journal.pone.0067742)
Supplement: Table S5 — (DOC) [file pone.0067742.s005.doc]

**Table S5.** Summary of genetic diversity of 41 foxtail accessions using 40 eSSR markers.

| **Sl. no.** | **Marker** | ***N_A_*** | **Allele size range (bp)** | ***H_o_*** | ***Nei (H_e_)*** | ***I*** | ***F*_IS_** | **PIC** |
| --- | --- | --- | --- | --- | --- | --- | --- | --- |
| 1 | SieSSR12 | 2 | 107-160 | 0.024 | 0.025 | 0.066 | -0.012 | 0.024 |
| 2 | SieSSR13 | 2 | 166-175 | 0.000 | 0.136 | 0.262 | 1.000 | 0.14 |
| 3 | SieSSR14 | 1 | 166 | 0.000 | 0.000 | 0.000 | - | 0.00 |
| 4 | SieSSR15 | 2 | 237-255 | 0.000 | 0.093 | 0.195 | 1.000 | 0.11 |
| 5 | SieSSR17 | 1 | 236 | 0.000 | 0.000 | 0.000 | - | 0.05 |
| 6 | SieSSR18 | 1 | 236 | 0.000 | 0.000 | 0.000 | - | 0.00 |
| 7 | SieSSR20 | 3 | 200-265 | 0.707 | 0.521 | 0.788 | -0.358 | 0.19 |
| 8 | SieSSR21 | 1 | 104 | 0.000 | 0.000 | 0.000 | - | 0.00 |
| 9 | SieSSR22 | 1 | 289 | 0.000 | 0.000 | 0.000 | - | 0.14 |
| 10 | SieSSR23 | 1 | 407 | 0.000 | 0.000 | 0.000 | - | 0.00 |
| 11 | SieSSR24 | 2 | 249-265 | 0.024 | 0.115 | 0.229 | 0.787 | 0.05 |
| 12 | SieSSR25 | 1 | 249 | 0.000 | 0.000 | 0.000 | - | 0.20 |
| 13 | SieSSR26 | 3 | 162-210 | 0.024 | 0.297 | 0.568 | 0.918 | 0.00 |
| 14 | SieSSR27 | 2 | 100-800 | 0.075 | 0.072 | 0.159 | -0.039 | 0.07 |
| 15 | SieSSR28 | 1 | 166 | 0.000 | 0.000 | 0.000 | - | 0.06 |
| 16 | SieSSR29 | 2 | 102-120 | 0.024 | 0.071 | 0.157 | 0.654 | 0.07 |
| 17 | SieSSR30 | 1 | 294 | 0.000 | 0.000 | 0.000 | - | 0.00 |
| 18 | SieSSR31 | 1 | 217 | 0.000 | 0.000 | 0.000 | - | 0.18 |
| 19 | SieSSR33 | 3 | 265-350 | 0.804 | 0.518 | 0.807 | -0.554 | 0.13 |
| 20 | SieSSR34 | 1 | 206 | 0.000 | 0.000 | 0.000 | - | 0.00 |
| 21 | SieSSR35 | 2 | 225-700 | 0.073 | 0.071 | 0.157 | -0.038 | 0.07 |
| 22 | SieSSR36 | 1 | 162 | 0.000 | 0.000 | 0.000 | - | 0.00 |
| 23 | SieSSR37 | 1 | 187 | 0.000 | 0.000 | 0.000 | - | 0.00 |
| 24 | SieSSR38 | 3 | 185-450 | 0.146 | 0.261 | 0.517 | 0.438 | 0.07 |
| 25 | SieSSR39 | 2 | 150-223 | 0.024 | 0.071 | 0.157 | 0.654 | 0.07 |
| 26 | SieSSR40 | 1 | 198 | 0.000 | 0.000 | 0.000 | - | 0.00 |
| 27 | SieSSR41 | 2 | 119-130 | 0.000 | 0.136 | 0.262 | 1.000 | 0.07 |
| 28 | SieSSR42 | 1 | 191 | 0.000 | 0.000 | 0.000 | - | 0.00 |
| 29 | SieSSR161 | 4 | 144-200 | 0.048 | 0.182 | 0.421 | 0.732 | 0.092 |
| 30 | SieSSR164 | 5 | 210-310 | 0.146 | 0.161 | 0.390 | 0.081 | 0.058 |
| 31 | SieSSR19c | 4 | 125-350 | 0.073 | 0.117 | 0.294 | 0.374 | 0.10 |
| 32 | SieSSR208 | 5 | 198-700 | 0.975 | 0.608 | 1.107 | -0.605 | 0.144 |
| 33 | SieSSR216 | 4 | 236-280 | 0.000 | 0.223 | 0.499 | 1.000 | 0.111 |
| 34 | SieSSR249 | 2 | 207-226 | 0.000 | 0.048 | 0.115 | 1.000 | 0.48 |
| 35 | SieSSR262 | 2 | 280-290 | 0.000 | 0.049 | 0.117 | 1.000 | 0.07 |
| 36 | SieSSR283 | 4 | 141-600 | 0.756 | 0.552 | 0.969 | -0.370 | 0.283 |
| 37 | SieSSR322b | 5 | 236-490 | 0.073 | 0.204 | 0.486 | 0.641 | 0.101 |
| 38 | SieSSR345 | 2 | 680-700 | 0.000 | 0.048 | 0.117 | 1.000 | 0.048 |
| 39 | SieSSR373 | 3 | 150-183 | 0.000 | 0.332 | 0.623 | 1.000 | 0.221 |
| 40 | SieSSR401 | 3 | 210-223 | 0.000 | 0.221 | 0.453 | 1.000 | 0.132 |
|  | **Mean**  **Std. Dev.** | **Total** = 88  2.2 allele/locus |  | 0.100  0.245 | 0.128  0.169 | 0.248  0.297 | 0.492 | 0.097 |

***N_A_*** -Number of alleles

***H_o_*** - Observed heterozygosity

***Nei (H_e_)*** - Nei’s average gene diversity

***I ­***- Shannon’s Informative Index

***F*_IS_** - Fixation index

**PIC** - Polymorphic information content
