# Supplementary material for: Development of eSSR-Markers in Setaria italica and Their Applicability in Studying Genetic Diversity, Cross-Transferability and Comparative Mapping in Millet and Non-Millet Species
Source: PLoS One. 2013 Jun 21;8(6):e67742. doi: 10.1371/journal.pone.0067742 (PMC3689721; doi:10.1371/journal.pone.0067742)
Supplement: Table S7 — (DOC) [file pone.0067742.s007.doc]

**Table S7.** Summary of comparative mapping between foxtail millet and sorghum using eSSR markers.

| **Foxtail Chromosomes (Total mapped markers)** | **Sorghum Chromosomes** | | | | | | | | | |
| --- | --- | --- | --- | --- | --- | --- | --- | --- | --- | --- |
|  | **SbChr1** | **SbChr2** | **SbChr3** | **SbChr4** | **SbChr5** | **SbChr6** | **SbChr7** | **SbChr8** | **SbChr9** | **SbChr10** |
| SiChr1 (25) | 2 | 0 | 2 | 17 | 0 | 1 | 1 | 0 | 1 | 1 |
| SiChr2 (21) | 0 | 20 | 0 | 0 | 1 | 0 | 0 | 0 | 0 | 0 |
| SiChr3 (25) | 1 | 0 | 1 | 1 | 0 | 2 | 0 | 5 | 15 | 0 |
| SiChr4 (18) | 0 | 1 | 0 | 0 | 0 | 1 | 0 | 0 | 0 | 16 |
| SiChr5 (29) | 0 | 0 | 27 | 1 | 0 | 1 | 0 | 0 | 0 | 0 |
| SiChr6 (16) | 2 | 0 | 0 | 1 | 0 | 1 | 12 | 0 | 0 | 0 |
| SiChr7 (26) | 0 | 0 | 0 | 0 | 0 | 15 | 0 | 8 | 1 | 2 |
| SiChr8 (10) | 1 | 0 | 0 | 1 | 4 | 1 | 0 | 3 | 0 | 0 |
| SiChr9 (53) | 51 | 0 | 0 | 0 | 2 | 0 | 0 | 0 | 0 | 0 |
| **Total (223)** | **57** | **21** | **30** | **21** | **7** | **22** | **13** | **16** | **17** | **19** |
