# Supplementary material for: Development of eSSR-Markers in Setaria italica and Their Applicability in Studying Genetic Diversity, Cross-Transferability and Comparative Mapping in Millet and Non-Millet Species
Source: PLoS One. 2013 Jun 21;8(6):e67742. doi: 10.1371/journal.pone.0067742 (PMC3689721; doi:10.1371/journal.pone.0067742)
Supplement: Table S8 — (DOC) [file pone.0067742.s008.doc]

**Table S8.** Summary of comparative mapping between foxtail millet and maize using eSSR markers.

| **Foxtail Chromosomes (Total mapped markers)** | **Maize Chromosomes** | | | | | | | | | |
| --- | --- | --- | --- | --- | --- | --- | --- | --- | --- | --- |
|  | **ZmChr1** | **ZmChr2** | **ZmChr3** | **ZmChr4** | **ZmChr5** | **ZmChr6** | **ZmChr7** | **ZmChr8** | **ZmChr9** | **ZmChr10** |
| SiChr1 (21) | 1 | 0 | 1 | 8 | 6 | 1 | 0 | 2 | 1 | 1 |
| SiChr2 (19) | 2 | 6 | 0 | 0 | 0 | 0 | 10 | 0 | 0 | 1 |
| SiChr3 (20) | 3 | 0 | 2 | 1 | 0 | 10 | 0 | 2 | 0 | 2 |
| SiChr4 (15) | 0 | 1 | 0 | 0 | 2 | 7 | 0 | 0 | 5 | 0 |
| SiChr5 (26) | 1 | 0 | 14 | 1 | 1 | 0 | 0 | 9 | 0 | 0 |
| SiChr6 (11) | 5 | 0 | 0 | 4 | 0 | 1 | 0 | 0 | 0 | 1 |
| SiChr7 (25) | 0 | 10 | 4 | 1 | 0 | 0 | 0 | 1 | 0 | 9 |
| SiChr8 (9) | 1 | 4 | 0 | 2 | 0 | 0 | 0 | 1 | 0 | 1 |
| SiChr9 (54) | 29 | 1 | 1 | 4 | 9 | 0 | 0 | 0 | 10 | 0 |
| **Total (200)** | **42** | **22** | **22** | **21** | **18** | **19** | **10** | **15** | **16** | **15** |
