# Supplementary material for: Development of eSSR-Markers in Setaria italica and Their Applicability in Studying Genetic Diversity, Cross-Transferability and Comparative Mapping in Millet and Non-Millet Species
Source: PLoS One. 2013 Jun 21;8(6):e67742. doi: 10.1371/journal.pone.0067742 (PMC3689721; doi:10.1371/journal.pone.0067742)
Supplement: Table S9 — (DOC) [file pone.0067742.s009.doc]

**Table S9.** Summary of comparative mapping between foxtail millet and rice using eSSR markers.

| **Foxtail Chromosomes (Total mapped markers)** | **Rice Chromosomes** | | | | | | | | | | | |
| --- | --- | --- | --- | --- | --- | --- | --- | --- | --- | --- | --- | --- |
|  | **OsChr1** | **OsChr2** | **OsChr3** | **OsChr4** | **OsChr5** | **OsChr6** | **OsChr7** | **OsChr8** | **OsChr9** | **OsChr10** | **OsChr11** | **OsChr12** |
| SiChr1 (13) | 0 | 11 | 0 | 0 | 0 | 0 | 0 | 0 | 1 | 0 | 0 | 1 |
| SiChr2 (14) | 1 | 0 | 0 | 0 | 0 | 1 | 7 | 0 | 4 | 0 | 0 | 1 |
| SiChr3 (17) | 2 | 0 | 1 | 1 | 7 | 0 | 1 | 0 | 1 | 1 | 0 | 3 |
| SiChr4 (8) | 0 | 0 | 0 | 0 | 0 | 7 | 1 | 0 | 0 | 0 | 0 | 0 |
| SiChr5 (16) | 13 | 1 | 1 | 0 | 1 | 0 | 0 | 0 | 0 | 0 | 0 | 0 |
| SiChr6 (9) | 0 | 0 | 1 | 0 | 0 | 0 | 0 | 6 | 2 | 0 | 0 | 0 |
| SiChr7 (16) | 0 | 1 | 0 | 11 | 1 | 0 | 0 | 0 | 0 | 0 | 1 | 2 |
| SiChr8 (9) | 4 | 0 | 0 | 1 | 0 | 0 | 0 | 0 | 0 | 0 | 4 | 0 |
| SiChr9 (34) | 1 | 0 | 22 | 0 | 0 | 1 | 1 | 0 | 0 | 6 | 2 | 1 |
| **Total (136)** | **24** | **13** | **24** | **12** | **9** | **9** | **10** | **6** | **8** | **6** | **7** | **8** |
